# Downregulation of FOXO3a by DNMT1 promotes breast cancer stem cell properties and tumorigenesis

**DOI:** 10.1038/s41418-019-0389-3

**Published:** 2019-07-11

**Authors:** Hao Liu, Ying Song, Huishi Qiu, Yanzhen Liu, Kai Luo, Yanmei Yi, Guanmin Jiang, Minying Lu, Zhijie Zhang, Jiang Yin, Shanshan Zeng, Xiangzhou Chen, Min Deng, Xiaoting Jia, Yixue Gu, Danyang Chen, Guopei Zheng, Zhimin He

**Affiliations:** 1 Affiliated Cancer Hospital and Institute of Guangzhou Medical University, Guangzhou Key Laboratory of “Translational Medicine on Malignant Tumor Treatment”, Guangzhou, Guangdong China; 2 Department of Radiation Oncology, The Sixth Affiliated Hospital of Guangzhou Medical University, Qingyuan, 511500 Guangdong China; 3 Guangdong Provincial Key Laboratory of Microbial Culture Collection and Application, Guangdong Institute of Microbiology, Guangzhou, 510070 China; 4 Department of Histology and Embryology, Guangdong medical university, Zhanjiang, Guangdong China; 5 Department of Clinical Laboratory, The Fifth Affiliated Hospital, Sun Yat-sen University, Zhuhai, Guangdong China

**Keywords:** Cancer stem cells, Tumour biomarkers

## Abstract

Breast cancer stem cells (BCSCs) are tumor initiating cells that can self-renew and are highly tumorigenic and chemoresistant. Therefore, the identification of factors critical for BCSC function is vital for the development of therapies. Here, we report that DNMT1-mediated FOXO3a promoter hypermethylation leads to downregulation of FOXO3a expression in breast cancer. FOXO3a is functionally related to the inhibition of FOXM1/SOX2 signaling and to the consequent suppression of BCSCs properties and tumorigenicity. Moreover, we found that SOX2 directly transactivates DNMT1 expression and thereby alters the methylation landscape, which in turn feedback inhibits FOXO3a expression. Inhibition of DNMT activity suppressed tumor growth via regulation of FOXO3a/FOXM1/SOX2 signaling in breast cancer. Clinically, we observed a significant inverse correlation between FOXO3a and FOXM1/SOX2/DNMT1 expression levels, and loss of FOXO3a expression or increased expression of FOXM1, SOX2, and DNMT1 predicted poor prognosis in breast cancer. Collectively, our findings suggest an important role of the DNMT1/FOXO3a/FOXM1/SOX2 pathway in regulating BCSCs properties, suggesting potential therapeutic targets for breast cancer.

## Introduction

Breast cancer is the most common cancer diagnosed and the second leading cause of cancer-related deaths among women worldwide [1]. Although early diagnosis and more effective treatment strategies have improved patient outcomes over the past few decades, a substantial portion of patients are refractory to current chemotherapeutic strategies. Breast cancer stem cells (BCSCs), a small subset of tumor cells with self-renewal ability, have been isolated from human breast cancers [2]. Because of their intrinsic stem cell-like properties, BCSCs play important roles in tumor progression and therapeutic resistance, and the ineffectiveness of conventional chemotherapy to eradicate BCSCs frequently result in therapy failure [3]. Therefore, understanding the regulation mechanisms of BCSCs might aid in the development of novel targeted strategies for eliminating BCSCs, thereby improving the clinical outcomes of patients with breast cancer.

Epigenetic programs contribute to gene expression regulation and have been proposed as key regulators of CSC self-renewal and differentiation [4]. Aberrant DNA methylation is one of the most common defects in epigenetic regulation observed in tumorigenesis [5]. Aberrant DNA hypermethylation at CpG islands, which leads to the loss of expression of genes specific to the differentiated state and regaining of stem cell-specific characteristics, has been reported to be critical for CSC properties in BCSCs [6]. CpG methylation is catalyzed by DNA methyltransferases (DNMTs), including DNMT1, DNMT3a, and DNMT3b [7]. A recent study uncovered an essential role for DNMTs in mammary stem/progenitor cell and BCSC maintenance [8]. DNMT deletion or inhibition of DNMT activity by a low dose of DNA demethylating agents (decitabine or azacitidine) has been shown to durably eradicate BCSCs [8–10]. However, the molecular mechanisms by which DNMTs regulate BCSCs remain largely elusive.

Forkhead box O3a (FOXO3a), a transcription factor of the FOXO protein family, has been highlighted as an important transcriptional regulator of crucial proteins associated with cell cycle progression, apoptosis, metastasis, angiogenesis, and metabolism [11–13]. Downregulation of FOXO3a leads to tumorigenesis, progression, and poor prognosis in many human cancers [14–16]. Interestingly, several studies have suggested that FOXO3a plays an important role in regulating CSC properties [17, 18]. For example, overexpression or pharmacological activation of FOXO3a inhibits stem-like properties and tumor initiation, and suppresses drug resistance in lung cancer cells and colorectal cancer [19, 20]. More recently, an integrated genomic approach revealed that FOXO3a is involved in breast cancer initiation [21]. However, the biological function and detailed molecular mechanism of FOXO3a in BCSCs are still unclear.

In the current study, we found that DNMT1-mediated FOXO3a promoter hypermethylation leads to downregulation of FOXO3a expression in breast cancer, and FOXO3a suppresses BCSC properties and tumorigenicity via inhibition of FOXM1/SOX2 signaling. Moreover, we demonstrated that SOX2 feedback inhibits FOXO3a expression by directly transactivating DNMT1, and inhibition of DNMT activity suppressed tumor growth via regulation of FOXO3a/FOXM1/SOX2 signaling in breast cancer. Our findings suggest an important role of DNMT1/FOXO3a/FOXM1/SOX2 signaling in regulating BCSC properties and establishes a strong rationale for developing therapeutic agents that target this pathway to control BCSCs and drug resistance.

## Results

### FOXO3a is downregulated and its promoter hypermethylated in breast cancer

**Fig. 1:**
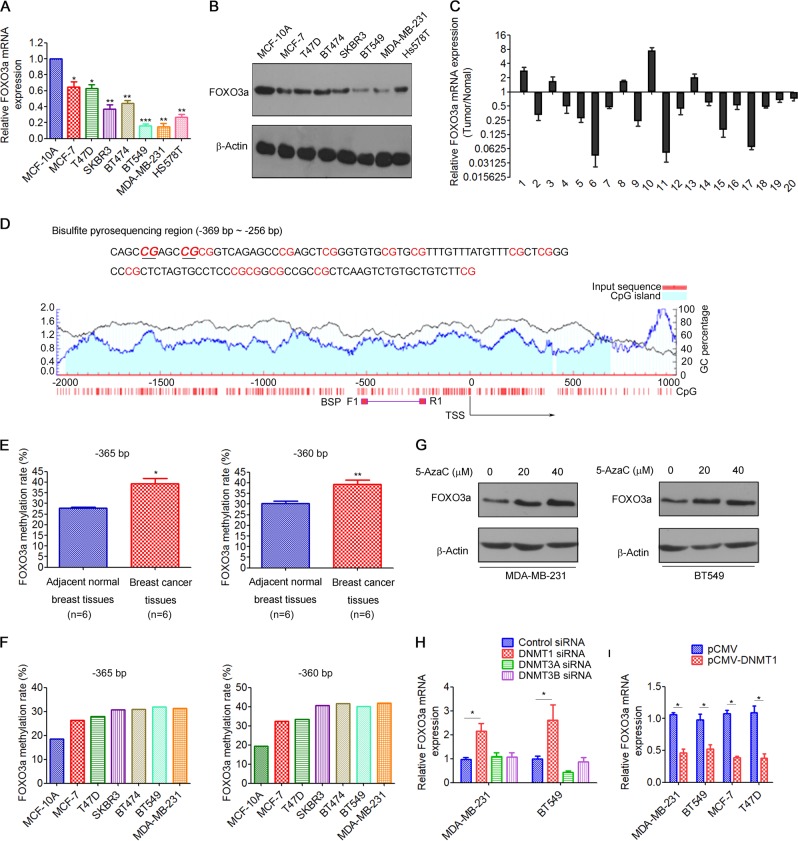
FOXO3a is downregulated and its promoter hypermethylated in breast cancer. **a**, **b** FOXO3a expression in the human breast epithelial cell line MCF-10A and a panel of breast cancer cell lines was measured by **a** qRT-PCR analysis and **b** western blot analysis. **c** Relative expression of FOXO3a in 20 pairs of breast cancer tissues (tumor) and corresponding adjacent normal breast tissues (normal). **d** Schematic representation of the CpG islands and bisulfite sequencing region in the FOXO3a promoter. Magenta words, CG sites for bisulfite sequencing; bold magenta words, the most significantly altered CG site in FOXO3a; Red region, input sequence; Blue region, CpG islands; Black curve, the trend of GC base percentage content; BSP F1 and R1, bisulfite forward primer and reverse primer. **e** Bisulfite sequencing analysis of the FOXO3a promoter region and the average methylation levels in normal (*n* = 6) and breast cancer (*n* = 6) tissues. **f** Methylation levels of the FOXO3a promoter region in MCF-10A cells and a panel of breast cancer cell lines. **g** MDA-MB-231 and BT549 cells were treated with 5-AzaC at indicated concentrations for 48 h, and FOXO3a expression was measured by western blot. **h** MDA-MB-231 and BT549 cells were transfected with DNMTs siRNA for 48 h, and FOXO3a mRNA expression was measured by qRT-PCR analysis. **i** Breast cancer cells were transfected with pCMV-DNMT1 for 48 h, and FOXO3a mRNA expression was measured by qRT-PCR analysis. A two-tailed Student’s *t* test was used for statistical analysis (*P < 0.05, **P < 0.01, ***P < 0.001)

Previous studies have demonstrated that FOXO3a is frequently downregulated in breast cancer [14]. Indeed, qRT-PCR and western blot results demonstrated that FOXO3a mRNA (Fig. 1a) and protein (Fig. 1b) levels were significantly downregulated in a panel of breast cancer cells compared to those in normal breast epithelial MCF-10A cells. Similarly, using mRNA expression data from 20 primary tumor samples, we found that FOXO3a mRNA levels were significantly decreased in tumor tissues than in adjacent normal tissues (Fig. 1c).

FOXO3a expression is regulated by the DNA methylation status of its promoter [22, 23]. Thus, we investigated whether the downregulation of FOXO3a was associated with the methylation status of its promoter in breast cancer. Bisulfite sequencing analysis was performed to examine FOXO3a promoter methylation levels in breast cancer tissues (*n* = 6) and normal tissues (*n* = 6). The CpG islands and the selected region for bisulfite sequencing in the FOXO3a promoter region are shown in Fig. 1d. We found that the methylation levels of two discrete CpG sites (−365 and +360 bp) in FOXO3a promoter were significantly increased in breast cancer tissues compared with that in the normal tissues (Fig. 1e and Fig. S1A). Similarly, FOXO3a methylation levels were substantially increased in the breast cancer cell lines compared with those in MCF10A cells (Fig. 1f). To determine whether the downregulation of FOXO3a resulted from its promoter hypermethylation, breast cancer cells were treated with the demethylation drug 5-AzaC. We found that treatment of 5-AzaC significantly increased the FOXO3a mRNA and protein levels in breast cancer cells (Fig. 1g and Fig. S1B). To establish the potential roles of the various DNMTs in mediating FOXO3a promoter methylation in breast cancer, we knocked down DNMT1, DNMT3A, and DNMT3B in breast cancer cells using specific small interfering RNAs (siRNAs) (Fig. S1C). Knockdown of DNMT1, but not DNMT3A and DNMT3B, resulted in restoration of FOXO3a expression (Fig. 1h and Fig. S1D). Moreover, overexpression of DNMT1 (Fig. S1E) significantly suppressed FOXO3a expression (Fig. 1i and Fig. S1F). To further determine the effects of DNA methylation on FOXO3a promoter activity and confirm the involvement of the two CpG sites (−365 and −360 bp) in promoter regulation, FOXO3a wild-type promoter constructs or promoter constructs containing site-specific CpG mutations were transfected into MCF-7 and T47D cells. Overexpression of DNMT1 significantly decreased the activity of the wild-type promoter, whereas mutations (CG to TG) created at the −365 or −360-bp CpG site reversed the inhibitory effect of DNMT1 on FOXO3a promoter activity, indicating that the methylation status of the two CpG sites (−365 or −360 bp) in the promoter region is pivotal in the epigenetic regulation of FOXO3a expression (Fig. S1G). Taken together, these findings suggested that the downregulation of FOXO3a is associated with hypermethylation of its promoter in breast cancer.

### FOXO3a suppresses BCSC properties in vitro

**Fig. 2:**
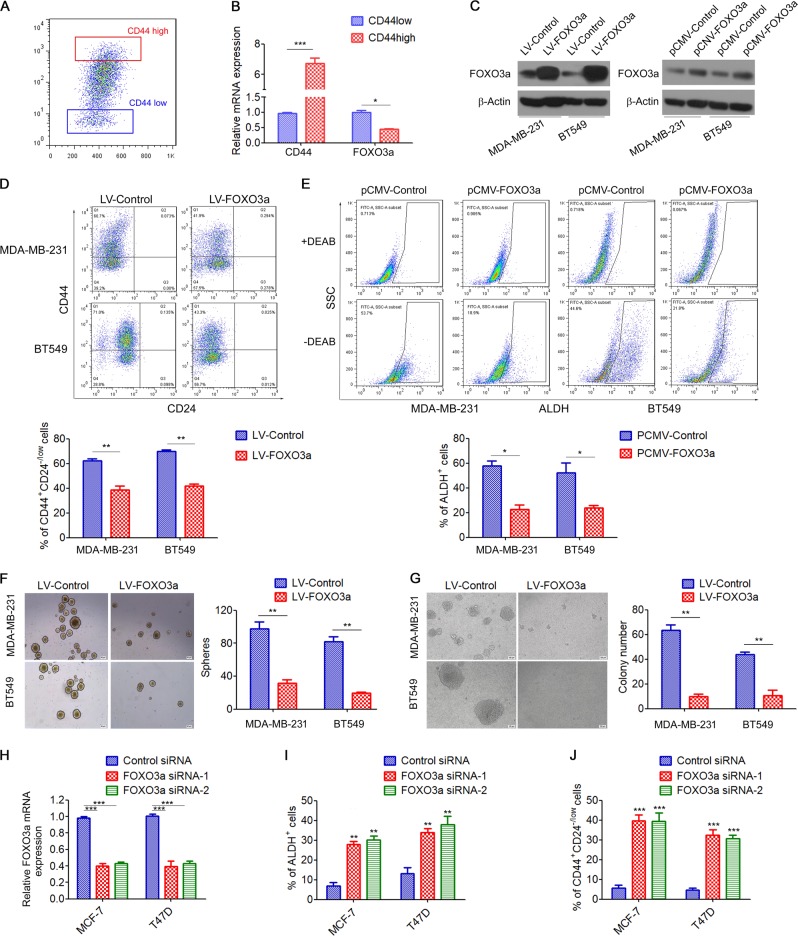
FOXO3a suppresses BCSC properties and tumorigenicity. **a** The top 10% of CD44^high^ or CD44^low^ subpopulations of MDA-MB-231 cells were isolated by flow cytometry sorting. **b** qRT-PCR analysis of FOXO3a expression in CD44^high^ or CD44^low^ subpopulations of MDA-MB-231 cells. **c** MDA-MB-231 and BT549 cells were transfected with FOXO3a-lentiviral vectors or pCMV-FOXO3a expression vector, and FOXO3a expression was measured by western blot. **d** MDA-MB-231 and BT549 cells were transfected with FOXO3a lentiviral vectors, and the percentages of CD44^high^/CD44^low^ cells were measured by flow cytometry. **e** MDA-MB-231 and BT549 cells were transfected with pCMV-FOXO3a expression vector, and the percentages of ALDH^+^ cells were measured by ALDEFLUOR assay. **f** Self-renewal of CSCs in control and FOXO3a-overexpressing cells as measured by a mammosphere formation assay. Scale bar, 50 μm. **g** Soft agar cloning in control and FOXO3a-overexpressing cells. Scale bar, 100 μm. **g**–**i** MCF-7 and T47D cells were transfected with FOXO3a siRNA, **h** FOXO3a expression was measured by qRT-PCR analysis; **i** the percentage of CD44^high^/CD44^low^ cells were measured by flow cytometry; **j** the percentage of ALDH^+^ cells was measured by ALDEFLUOR assay. A two-tailed Student’s *t* test was used for statistical analysis (*P < 0.05, **P < 0.01, ***P < 0.001)

Human BCSCs are commonly characterized in vitro by expression of CD44^high^/CD24^−/low^ surface markers [2]. Previous studies have shown that triple negative breast cancer (TNBC) cells contain a high proportion of the CD44^high^/CD24^−/low^ BCSC population, whereas ER^+^ breast cancer cells contain a low proportion of the CD44^high^/CD24^−/low^ BCSC population [24, 25]. As FOXO3a was more strongly expressed in ER^+^ breast cancer cells (MCF7 and T47D) than in TNBC cells (MDA-MB-231 and BT549) (Fig. 1a, b), we then investigated whether downregulation of FOXO3a plays a key role in BCSC properties. We isolated the 10% of cells with the highest (CD44^high^) or lowest (CD44^low^) CD44 expression from MDA-MB-231 cells (Fig. 2a), and found that FOXO3a expression was significantly decreased in CD44^high^ cells compared to CD44^low^ cells (Fig. 2b). We next overexpressed FOXO3a in MDA-MB-231 and BT549 cells using lentiviral vectors or pCMV vector (pCMV-FOXO3a) (Fig. 2c). Flow cytometry analysis revealed that overexpression of FOXO3a led to a significant reduction in the CD44^+^/CD24^−^ subpopulation in BT549 and MDA-MB-231 cells (Fig. 2d). Enhanced aldehyde dehydrogenase (ALDH) activity is another hallmark of BCSCs [26]. Indeed, ALDEFLUOR assay revealed that transient transfection of the breast cancer cells with pCMV-FOXO3a (Fig. 2c) significantly decreased the percentages of ALDH^+^ cells (Fig. 2e). Consistently, overexpression of FOXO3a effectively suppressed mammosphere formation (Fig. 2f) and anchorage-independent growth (Fig. 2g). In contrast, stable knockdown of FOXO3a by either of two short hairpin RNAs (shRNAs) (Fig. S2A) significantly increased the mammosphere formation capacity (Fig. S2B). Moreover, siRNA-mediated FOXO3a knockdown (Fig. 2h and Fig. S2C) resulted in markedly increased percentages of ALDH^+^ cells (Fig. 2i and Fig. S2D), as well as increased CD44^+^/CD24^−^ cells population (Fig. 2j and Fig. S2E) in MCF-7 and T47D cells. Furthermore, stable downregulation of FOXO3a expression in MCF-7 and T47D cells enhanced their resistance to the antiproliferative effects of the chemotherapy drugs doxorubicin and paclitaxel (Fig. S3). Collectively, these results demonstrated that downregulation of FOXO3a is essential for the maintenance of the increased CSC population associated with the development of drug resistance in breast cancer.

### FOXO3a impairs tumorigenicity and tumor growth in vivo

**Fig. 3:**
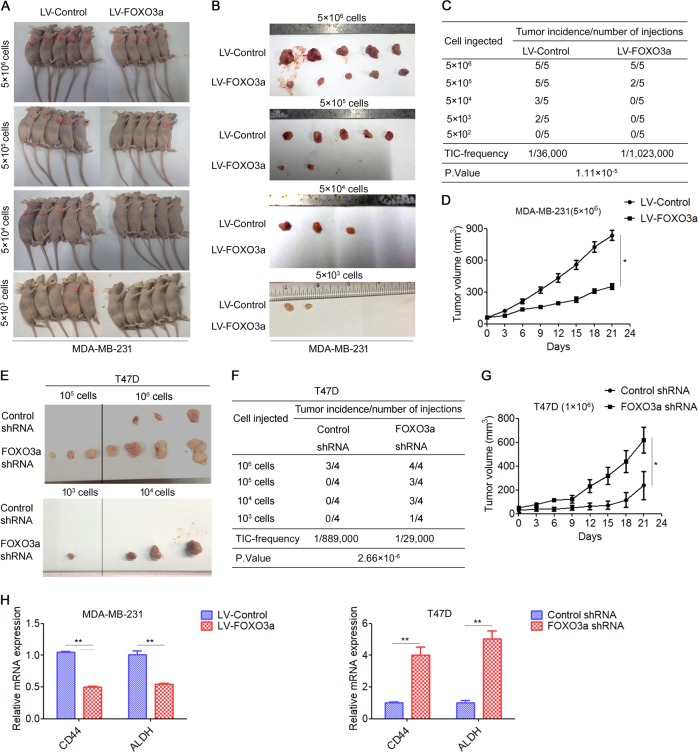
FOXO3a impairs tumorigenicity and tumor growth in vivo. **a**–**d** 5 × 10^6^, 5 × 10^5^, 5 × 10^4^, 5 × 10^3^, or 5 × 10^2^ MDA-MB-231/control and MDA-MB-231/FOXO3a cells were implanted in nude mice (*n* = 5 per group), **a** Representative images of the tumors are shown; **b** At the experimental endpoint, the tumors were dissected and imaged as indicated; **c** Tumor formation frequencies for different numbers of the indicated cells; **d** The tumor volume was measured on the indicated days. **e**, **f** 1 × 10^6^, 1 × 10^5^, 1 × 10^4^, or 1 × 10^3^ T47D/control shRNA and T47D/FOXO3a shRNA cells were implanted in nude mice (*n* = 4 per group), **e** At the experimental endpoint, the tumors were dissected and imaged as indicated; **f** Tumor formation frequencies for different numbers of the indicated cells. **g** The tumor volume was measured on the indicated days. **h** qRT-PCR detection of CD44 and ALDH1 expression in the indicated tumor tissues. A two-tailed Student’s *t* test was used for statistical analysis (*P < 0.05, **P < 0.01)

Tumor-initiating ability is another criterion for CSC properties [27]. Thus, we analyzed the role of FOXO3a in the tumor-initiating potential of breast cancer cells. Five doses (5 × 10^6^, 5 × 10^5^, 5 × 10^4^, 5 × 10^3^, and 5 × 10^2^) of FOXO3a-overexpressing MDA-MB-231 cells or control cells were subcutaneously injected in nude mice (Fig. 3a). We found that FOXO3a-overexpressing cells showed a striking 28-fold reduction in tumor-initiating cell (TIC) frequency compared to control cells (Fig. 3b, c). Moreover, overexpression of FOXO3a also significantly inhibited tumor growth (Fig. 3d). Consistently, FOXO3a-overexpressing BT549 cells displayed lower tumorigenicity and lower tumor growth rates than control cells (Fig. S4A–D). In contrast, FOXO3a-knockdown T47D and MCF-7 cells showed increased tumorigenicity and faster growth, and formed larger tumors than control cells (Fig. 3e–g, and Fig. S4E–F). In addition, tumors formed by FOXO3a-overexpressing cells showed obviously downregulated levels of CD44 and ALDH1 (Fig. 3h), whereas tumors formed by FOXO3a-knockdown cells showed upregulated levels of CD44 and ALDH1 (Fig. 3h). These results indicated that FOXO3a significantly impairs tumorigenicity and tumor growth in breast cancer.

### FOXO3a suppresses BCSC properties via inhibition of FOXM1

**Fig. 4:**
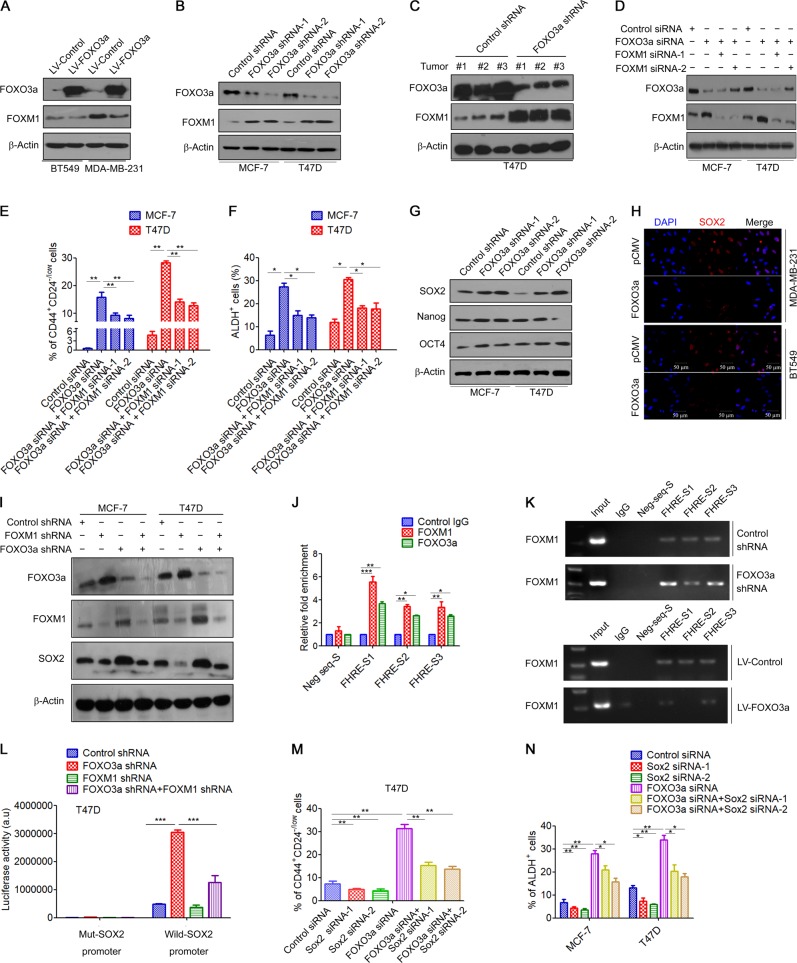
FOXO3a suppresses BCSC properties via inhibition of FOXM1/SOX2 signaling. **a** MDA-MB-231 and BT549 cells were transfected with FOXO3a lentiviral vectors, and FOXO3a and FOXM1 expression was measured by western blot. **b** MCF-7 and T47D cells were transfected with FOXO3a shRNA, FOXO3a and FOXM1 expression was measured by western blot. **c** FOXO3a and FOXM1 expression in T47D tumor xenografts as analyzed by western blot. **d**–**f** MCF-7 and T47D cells were transfected with FOXO3a siRNA plus FOXM1 siRNA, **d** FOXO3a and FOXM1 expression was measured by Western blot; **e** The percentage of CD44^high^/CD44^low^ cells was measured using flow cytometry; **f** The percentage of ALDH^+^ cells was measured by ALDEFLUOR assay. **g** MCF-7 and T47D cells were transfected with FOXO3a shRNA, SOX2, and OCT4, and Nanog expression was measured by western blot. **h** MDA-MB-231 and BT549 cells were transfected with FOXO3a-expression vector (pCMV-FOXO3a), cells were fixed in 4% formaldehyde, and SOX2 was visualized with rabbit monoclonal antibody followed by the addition of secondary anti-rabbit antibody conjugated to PE (red). Nuclei were counterstained with DAPI (blue). Scale bar, 50 μm. **i** MCF-7 and T47D cells were transfected with FOXO3a siRNA plus FOXM1 siRNA, FOXO3a and FOXM1 expression was measured by western blot. **j** ChIP assay using chromatin prepared from MDA-MB-231 cells to analyze SOX2 promoter occupation by FOXO3a and FOXM1. The chromatin was precipitated with the anti-FOXO3a antibody or anti-FOXM1 antibody or control (IgG). The precipitated chromatin was then analyzed by qRT-PCR with primers specific for the putative FOX consensus binding sites or a control region. **k** MCF-7 cells were transfected with FOXO3a shRNA, or MDA-MB-231 cells were transfected with FOXO3a-lentiviral vectors, and the chromatin was precipitated with the anti-FOXM1 antibody or control (IgG). The precipitated chromatin was then analyzed by qRT-PCR and resolved in a 2% agarose gel. **l** T47D cells were transfected with FOXO3a shRNA or/and FOXM1 shRNA together with a luciferase reporter construct containing the wild-type or indicated mutant promoter regions. Relative luciferase activities were measured 48 h after transfection. Firefly luciferase activity of the reporter construct was normalized to internal Renilla luciferase activity. **m** T47D cells were transfected with FOXO3a siRNA plus SOX2 siRNA, the percentage of CD44^high^/CD44^low^ cells was measured using flow cytometry. **n** MCF-7 and T47D cells were transfected with FOXO3a siRNA plus SOX2 siRNA, the percentage of ALDH^+^ cells was measured by ALDEFLUOR assay. A two-tailed Student’s *t* test was used for statistical analysis (*P < 0.05, **P < 0.01, ***P < 0.001)

Recent studies have suggested that FOXM1 plays a critical role in maintaining the CSC self-renewal and tumorigenic potential [28], and FOXO3a can bind to the promoter of FOXM1 to suppress its transcription [29, 30]. Therefore, we evaluated whether FOXO3a affects BCSC properties by controlling FOXM1 signaling. Western blot analysis revealed that overexpression of FOXO3a significantly suppressed FOXM1 protein expression in MDA-MB-231 and BT549 cells (Fig. 4a). These results were verified by immunofluorescence analysis (Fig. S5A–B). Furthermore, knockdown of FOXO3a significantly increased the expression of FOXM1 in MCF-7 and T47D cells (Fig. 4b and Fig. S5C). Elevated FOXM1 protein levels were also observed in FOXO3a-knockdown tumor xenografts (Fig. 4c).

We next examined whether the FOXO3a/FOXM1 axis regulates the stemness of BCSCs. To this end, FOXO3a-knockdown MCF7 and T47D cells were transfected with FOXM1 siRNA (Fig. 4d). The results showed that the positive effects of FOXO3a knockdown on the CD44^+^/CD24^−^ cells population and ALDH^+^ cells percentages were significantly impaired by FOXM1 depletion (Fig. 4e, f and Fig. S6A–B). Moreover, transfection of FOXM1 shRNA significantly decreased the mammosphere formation capacity and anchorage-independent growth in FOXO3a-knockdown cells (Fig. S6C–D). Together, these data supported the notion that FOXM1 contributes to FOXO3a-mediated BCSCs inhibition.

### FOXO3a/FOXM1 axis regulates BCSC properties via a SOX2-dependent mechanism

To gain insight into how the FOXO3a/FOXM1 axis regulates stem-like properties of breast cancer cells, we first examined the expression of five stemness-related genes after overexpressing FOXO3a or downregulating FOXO3a in MCF-7 and T47D cells. qRT-PCR results showed that SOX2 expression was differentially affected in both conditions (Fig. S7A–B). These results were further verified by western blot and immunofluorescence analysis, which showed that knockdown of FOXO3a significantly increased SOX2 protein levels in both MCF-7 and T47D cells (Fig. 4g and Fig. S7C), whereas overexpression of FOXO3a significantly reduced SOX2 protein levels in both MDA-MB-231 and BT549 cells (Fig. 4h). Importantly, the increases in SOX2 mRNA and protein levels induced by FOXO3a knockdown were abrogated upon cotransfection with FOXM1 shRNA (Fig. 4i and Fig. S7D), suggesting that FOXO3a inhibited SOX2 expression in a FOXM1-dependent manner.

We further elucidated how the FOXO3a/FOXM1 axis regulates SOX2 expression. Sequence analysis of the SOX2 promoter revealed three conserved FOX-binding sites at the core promoter region (FHRE-S1, FHRE-S2, and FHRE-S3) (Fig. S8A). To validate a direct binding of FOXO3a or FOXM1 to the SOX2 promoter region, we conducted a chromatin immunoprecipitation (ChIP)-qPCR assay in MDA-MB-231 cells using anti-FOXO3a and anti-FOXM1 antibody, respectively. Both FOXO3a and FOXM1 could bind to all three FHRE regions but not the control region (Fig. 4j). A previous study suggested that FOXO3a can antagonize FOXM1 function by competing for the same target genes [31]. Indeed, we found that knockdown of FOXO3a resulted in increased FOXM1 binding to the FHREs, whereas overexpression of FOXO3a significantly suppressed FOXM1 binding to FHREs (Fig. 4k). To further demonstrate the regulatory action of FOXO3a/FOXM1 on SOX2 promoter, we conducted a luciferase reporter assay. Our results showed that luciferase expression directed by a 1960-bp fragment of the SOX2 promoter containing the FHREs was decreased in FOXO3a-overexpressing BT549 cells (Fig. S8B). In contrast, knockdown of FOXO3a resulted in a significant increase in SOX2 promoter activity in MCF-7 and T47D cells (Fig. 4l and Fig. S8C). In addition, transfection of FOXM1 shRNA significantly decreased SOX2 promoter activity in FOXO3a-knockdown cells (Fig. 4l and Fig. S8C). These results suggested that FOXO3a and FOXM1 bind directly to the SOX2 promoter, and have opposite effects on SOX2 promoter transactivation.

To validate that SOX2 was responsible for FOXO3a/FOXM1-mediated inhibition of BCSC properties, we utilized siRNA targeting SOX2 (Fig. S9A). We found that transfection with SOX2 siRNA significantly decreased the CD44^+^/CD24^−^ cells population and ALDH^+^ cells percentages in FOXO3a-knockdown cells (Fig. 4m, n). Moreover, enhanced mammosphere formation capacity and anchorage-independent growth induced by FOXO3a shRNA were reversed by SOX2 siRNA (Fig. S9B–C). Together, these results suggested that the regulation of CSC properties by the FOXO3a/FOXM1 axis is mediated by SOX2 in breast cancer cells.

### SOX2 feedback inhibits FOXO3a expression by activating DNMT1

**Fig. 5:**
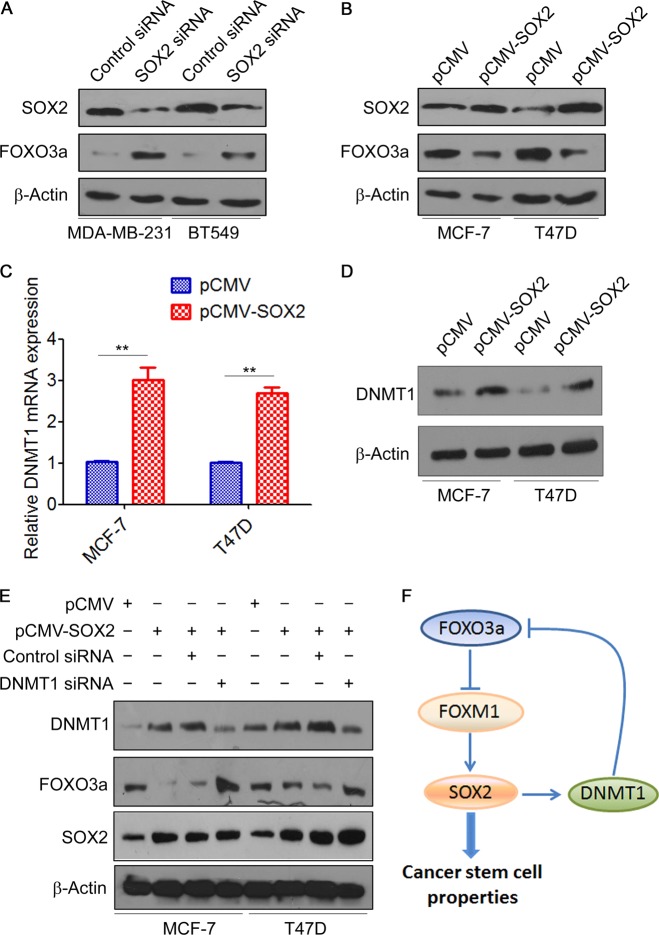
SOX2 feedback inhibits FOXO3a expression by activating DNMT1. **a** MDA-MB-231 and BT549 cells were transfected with SOX2 siRNA, SOX2 and FOXO3a expressions were measured by western blot. **b** MCF-7 and T47D cells were transfected with SOX2 expression vector, SOX2 and FOXO3a expression was measured by western blot. **c**, **d** MCF-7 and T47D cells were transfected with SOX2 expression vector, DNMT1 expression was measured by qRT-PCR (**c**) and western blot (**d**). A two-tailed Student’s *t* test was used for statistical analysis. (**P < 0.01). **e** MCF-7 and T47D cells were transfected with SOX2 expression vector plus DNMT1 siRNA, the expressions of DNMT1, SOX2, and FOXO3a were measured by western blot. **f** Illustration of SOX2 feedback inhibits FOXO3a expression by activating DNMT1

As we observed a significant increase in FOXO3a mRNA expression after depletion of SOX2 (Fig. S9A), we speculated that FOXO3a might be regulated by SOX2. Indeed, western blot analysis confirmed that knockdown of SOX2 increased the protein expression of FOXO3a in MDA-MB-231 and BT549 cells (Fig. 5a), whereas overexpression of SOX2 decreased the protein expression of FOXO3a in MCF-7 and T47D cells (Fig. 5b). SOX2 reportedly binds to the DNMT1 promoter to induce DNMT1 expression [32]. Therefore, we hypothesized that SOX2 directly transactivates DNMT1 expression and thereby alters the methylation landscape and inhibits FOXO3a expression in breast cancer. Indeed, overexpression of SOX2 significantly increased the mRNA and protein levels of DNMT1 (Fig. 5c, d). Furthermore, FOXO3a inhibition by SOX2-overexpressing vector was completely abolished by transfection with DNMT1 siRNA (Fig. 5e). Together, these results suggested that SOX2 feedback inhibits FOXO3a expression by activating DNMT1 (Fig. 5f).

### Dysregulation of DNMT1/FOXO3a/FOXM1/SOX2 signaling exhibits clinical significance

**Fig. 6:**
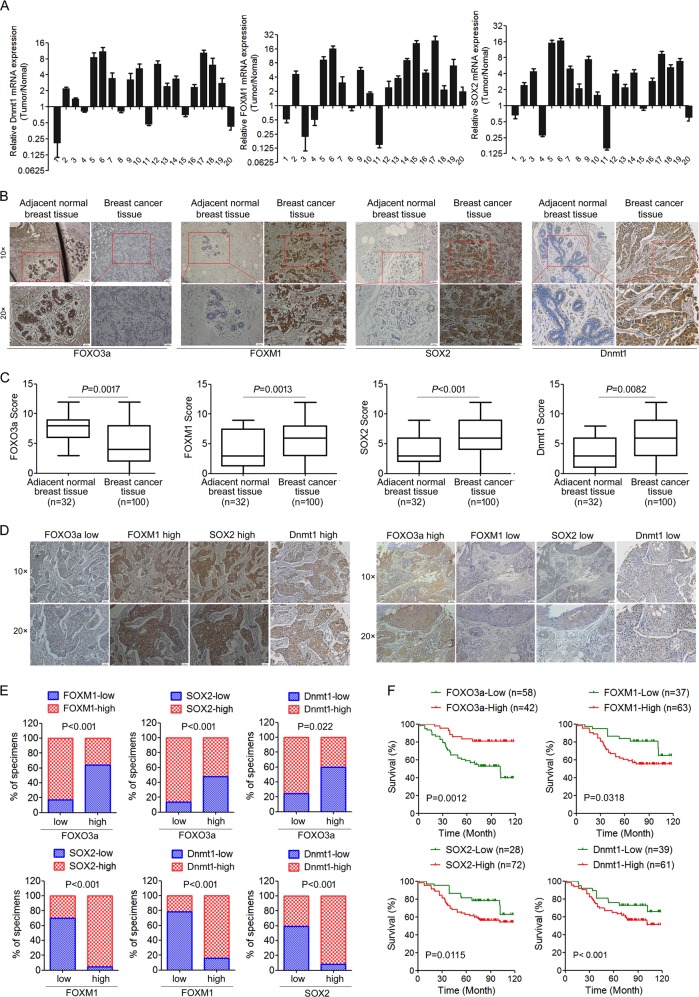
Dysregulation of DNMT1/FOXO3a/FOXM1/SOX2 signaling exhibits clinical significance. **a** Relative expression of FOXM1, SOX2, and DNMT1 in 20 pairs of breast cancer tissues (tumor) and their corresponding adjacent normal breast tissues (normal). **b** Representative immunohistochemical staining of FOXO3a, FOXM1, SOX2, and DNMT1 protein expression in breast cancer tissue specimens. **c** FOXO3a, FOXM1, SOX2, and DNMT1 expression scores in breast cancer tissue specimens. The breast cancer tissue sections were quantitatively scored according to the percentage of positive cells and staining intensity as described in the “Materials and Methods.” **d** Representative examples of the FOXO3a, FOXM1, SOX2, and DNMT1 staining in the same breast cancer tissue set. (left) FOXO3a^low^/FOXM1^high^/SOX2^high^/Dnmt1^high^; (right) FOXO3a^high^/ FOXM1^low^/SOX2^low^/Dnmt1^low^. **e** Clinical correlations among the network of regulatory genes, FOXO3a, FOXM1, SOX2, and DNMT1 in breast cancer samples. **f** Survival curves of breast cancer patients with low expression versus high expression of FOXO3a, FOXM1, SOX2, and DNMT1

We further investigated the clinical significance of DNMT1/FOXO3a/FOXM1/SOX2 signaling in breast cancer. Using mRNA expression data from 20 primary tumor samples, we found that the mRNA expression levels of DNMT1, FOXM1, and SOX2 were significantly increased in tumor tissues (Fig. 6a). Moreover, we compared FOXO3a, FOXM1, SOX2, and DNMT1 expression in a tissue microarray containing 100 independent primary breast tumor samples and 32 adjacent normal breast tissues by immunohistochemistry. We found that, in general, adjacent normal tissues exhibited relatively high FOXO3a expression and very low expression of FOXM1, SOX2, and DNMT1; in contrast, breast cancer tissues had low FOXO3a, but high FOXM1, SOX2, and DNMT1 expression (Fig. 6b, c). Importantly, we observed a significant inverse correlation between FOXO3a and FOXM1/SOX2/DNMT1 expression levels, and a significant positive correlation between FOXM1, SOX2, and DNMT1 in the breast cancer tissue set (Fig. 6d, e and Fig. S10), which was consistent with our finding in vitro and in animal model. Next, we analyzed the clinicopathological implication of FOXO3a, FOXM1, SOX2, and DNMT1 levels in breast cancer patients. Correlations between FOXO3a expression and various clinicopathological characteristics are summarized in Table S4. FOXO3a expression was not correlated with patient age. However, statistically significant negative correlations were found between FOXO3a expression and histological grade (*P* = 0.0268), advanced TNM stage (*P* = 0.0211), and lymph node metastasis (*P* < 0.01). Moreover, high levels of FOXM1, SOX2, and DNMT1 were correlated with high histological grade, advanced clinical stage, and lymph node metastasis (Table S5). Moreover, 60% (31/51) of tumors expressed moderate or high levels of FOXO3a in luminal subtypes, whereas 63% (12/19) and 90% (27/30) of tumors expressed low levels of FOXO3a in HER2+ and TNBC subtypes, respectively (Table S4). In contrast, substantially higher protein levels of FOXM1 and SOX2 were observed in HER2+ and TNBC subtypes (Table S5). However, DNMT1 expression was not significantly different among the subtypes (Table S5). Furthermore, we examined whether the levels of FOXO3a, FOXM1, SOX2, and DNMT1 were associated with the survival of patients with breast cancer. Kaplan–Meier survival analyses revealed that patients with low FOXO3a expression had poorer overall survival than patients with high FOXO3a expression, whereas patients with high FOXM1, SOX2, or DNMT1 expression had poorer overall survival than patients with low FOXM1, SOX2, or Dnmt1 expression (Fig. 6f). We further evaluated the prognostic value of combined use of the four biomarkers. We found that the combination of low FOXO3a expression and high FOXM1, SOX2, and DNMT1 expression was a strong predictor of shorter survival in breast cancer patients (Fig. S11A). The prognostic values of FOXO3a, FOXM1, SOX2, and DNMT1 were further validated at the mRNA level in the cases from the Kaplan–Meier plotter dataset. Similar finding was obtained supporting the prognostic value of FOXO3a, FOXM1, SOX2, and DNMT1 in the whole cohort (Fig. S11B). We next analyzed the correlation of FOXO3a, FOXM1, SOX2, and DNMT1 expression to the prognosis of breast cancer patients with lymph node metastasis status. Lower levels of FOXO3a, and higher levels of FOXM1, SOX2, or DNMT1 were correlated with shorter survival in the lymph node metastasis positive subgroup. However, no significant difference in prognosis was observed between lymph node metastasis negative breast cancer patients who have either high or low FOXO3a (Fig. S11B). As for the various molecular typing groups, low FOXO3a expression, or high expression of FOXM1, SOX2, and DNMT1 was relevant to shorter survival in ERα+ breast cancer patients. In TNBC, we also observed a similar trend, but the correlation did not reach statistical significance (Fig. S11B). Taken together, these findings indicated that dysregulated FOXO3a/FOXM1/SOX2/DNMT1 signaling plays a critical role in disease progression and is a valuable biomarker in breast cancer.

### Inhibition of DNMT activity suppresses tumorigenesis and tumor growth via regulation of FOXO3a/FOXM1/SOX2 signaling

**Fig. 7:**
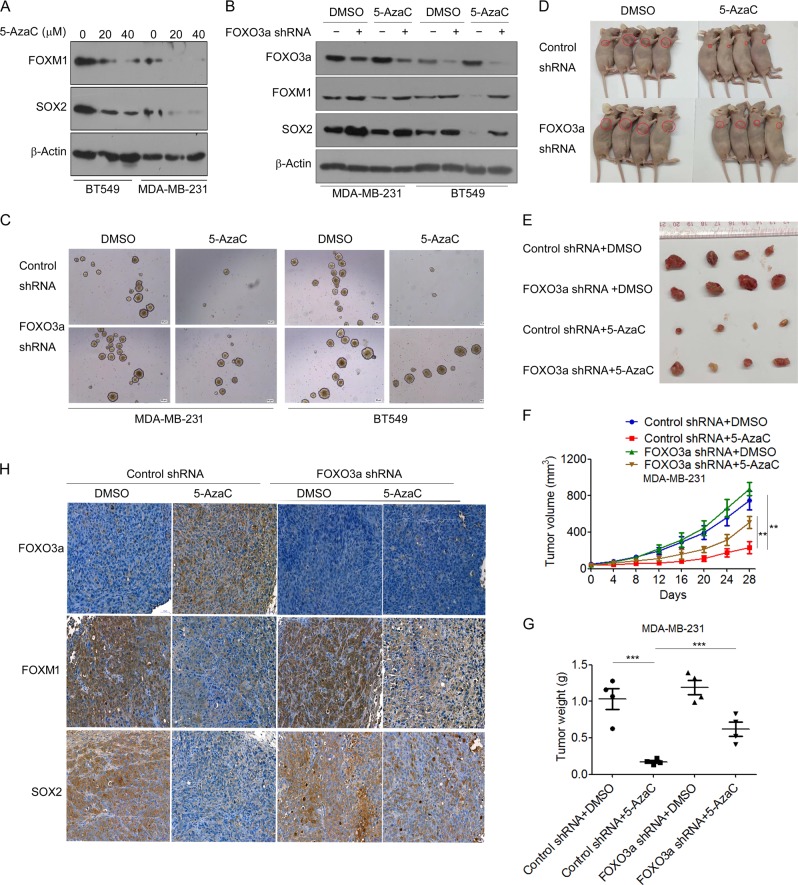
Inhibition of DNMT activity suppresses tumorigenesis and tumor growth via regulation of FOXO3a/FOXM1/SOX2 signaling. **a** MDA-MB-231 and BT549 cells were treated with 5-AzaC at indicated concentrations for 48 h, FOXM1 and SOX2 expression was measured by western blot. **b**, **c** MDA-MB-231 and BT549 cells were transfected with FOXO3a shRNA, then treated with 5-AzaC, **b** the expression of FOXO3a, FOXM1, and SOX2 was measured by western blot; **c** Mammosphere formation was measured. **d**–**g** MDA-MB-231 cells tranfected with FOXO3a shRNA or control shRNA were implanted in nude mice and palpable tumors were allowed to develop for 7 days. 5-AzaC at a dose of 0.5 mg/kg body weight was injected every other day for 4 weeks, **d** Representative images of the tumors are shown; **e** At the end of treatment, tumors were excised and subjected to further analyses; **f** Tumor sizes were measured at indicated time intervals; **g** Tumor weights were measured. **h** Tumor tissues derived from mice were resected, fixed, sectioned, and placed on slides. Tumor specimens were subjected to immunohistochemical staining with antibodies specific to FOXO3a, FOXM1, and SOX2. A two-tailed Student’s *t* test was used for statistical analysis. (**P < 0.01, ***P < 0.01)

Because DNMT1-mediated methylation downregulated FOXO3a expression (Fig. 1), we next tested whether pharmacological inhibition of DNMTs could suppress tumorigenesis and tumor growth by regulating FOXO3a/FOXM1/SOX2 signaling. We found that, along with increased expression of FOXO3a (Fig. 1g), treatment with 5-AzaC significantly inhibited the expression of FOXM1 and SOX2 (Fig. 7a). We next sought to determine whether upregulation of FOXO3a participated in 5-AzaC-mediated inhibition of FOXM1 and SOX2. MDA-MB-231 and BT549 cells were transfected with FOXO3a shRNA to stably knockdown FOXO3a (Fig. 7b), and were then treated with 5-AzaC. Knockdown of FOXO3a led to a significant increase in the expression of FOXM1 and SOX2 in 5-AzaC-treated cells (Fig. 7b). Moreover, treatment with 5-AzaC decreased the mammosphere formation potential in BT549 and MDA-MB-231 cells, which was reversed by FOXO3a shRNA transfection (Fig. 7c). Furthermore, we examined the ability of 5-AzaC to suppress the growth of MDA-MB-231 and BT549 tumor xenografts in nude mice. We found that treatment with 5-AzaC significantly inhibited the growth of MDA-MB-231 tumor xenografts (Fig. 7d–g). The inhibitory effect of 5-AzaC on tumor growth was further verified in BT549 tumor xenografts (Fig. S12A–D). Knockdown of FOXO3a reversed the inhibitory effect of 5-AzaC on MDA-MB-231 tumor xenograft growth (Fig. 7d–g). Furthermore, immunohistochemical staining revealed that 5-AzaC treatment significantly enhanced FOXO3a expression, but decreased FOXM1 and SOX2 expression in xenograft tumors (Fig. 7h and Fig. S12E). Taken together, these data showed that inhibition of DNMT activity suppresses tumor growth via regulating FOXO3a/FOXM1/SOX2 signaling in breast cancer.

## Discussion

CSCs play important roles in tumorigenesis, chemoresistance, and tumor recurrence, and the ineffectiveness of conventional chemotherapy to eradicate CSCs frequently results in therapy failure [33]. To date, the role of FOXO3a in CSCs is controversial. Some studies reported that knockdown of FOXO3a led to expansion of the CSC population as well as increased self-renewal and tumorigenic capacity in lung and breast cancer [17, 18, 21], and that FOXO3a activation could inhibit CSC properties and tumor initiation in colorectal cancer [20]. However, other studies reported that FOXO3a knockdown suppressed CD44 expression and CSC properties in pancreatic cancer cells [34, 35]. The contrasting roles of FOXO3a in the maintenance of CSC properties documented suggest that FOXO3a might have different functions in CSCs of different cancer types. In this study, we demonstrated that FOXO3a inhibited mammosphere formation ability, percentages of ALDH^+^ cells, subpopulation of CD44^+^/CD24^−^ cells in vitro, and tumorigenicity in vivo, which supported an important role of FOXO3a in inhibiting CSC properties of breast cancer cells. Moreover, FOXO3a has been shown to inhibit breast cancer proliferation through transcriptional regulation of multiple proteins, including p21Cip1, p27Kip1, and cyclin D1 [36]. Indeed, the current study showed that FOXO3a not only affected tumorigenesis, but also inhibited tumor growth. These results are consistent with recent findings that overexpression of FOXO3a can suppress tumorigenesis and proliferation in athymic mice [37], which suggest that FOXO3a plays multiple roles in the progression of breast cancer.

FOXO3a and FOXM1 are two forkhead transcription factors with antagonistic roles in cancer progression [38]. FOXO3a functions as a typical tumor suppressor, whereas FOXM1 is a potent oncogene [31]. The importance of FOXM1 in the development of stem cell-like properties has been well defined in various cancer types [39–42]. High FOXM1 expression was detected in CSCs and TICs. FOXM1 promoted the proliferation and maintenance of breast [28, 43], hepatocellular [44], pancreatic [45], and lung CSCs [46], and FOXM1 depletion repressed the stemness of these cancer cells. FOXM1 is an important component of the reprogramming network and functions together with reprogramming transcriptional factors, such as OCT4, SOX2, and KLF4, to regulate CSC self-renewal and maintenance [47, 48]. For example, FOXM1 has been shown to promote stem cell pluripotency by promoting the transcription of OCT4, which helps to suppress cellular differentiation [49]. FOXM1 has been shown to bind directly to the SOX2 promoter to induce its expression [50]. Multiple studies have demonstrated that FOXO3a not only directly inhibits FOXM1 transcription, but also antagonizes FOXM1 function by competing for the same target genes [29, 30]. Based on these observations, we speculated that FOXO3a might affect BCSC properties by controlling FOXM1 signaling. Indeed, we found that FOXO3a significantly decreased FOXM1 expression, and inhibited SOX2 expression in a FOXM1-dependent manner. This result supports a critical role of the FOXO3a/FOXM1/SOX2 pathway in regulating CSC properties in breast cancer. However, we found that SOX2 silencing did not fully rescue the CSC phenotype induced by FOXO3 silencing, suggesting that other pathways might be modulated by FOXO3 to induce stemness in these models. This remains to be evaluated in future studies.

Loss of FOXO3a has been observed in various cancers [14, 51], and its cellular localization and phosphorylation status are considered to be prognostic factors for acute myeloid leukemia [52], breast [15, 53], prostate [54], and ovarian cancer [55]. FOXO3a is primarily regulated by posttranslational mechanisms. Multiple kinases, including AKT, MAPK, and GSK, can phosphorylate FOXO3a, which leads to nuclear exclusion and ubiquitination/degradation [14, 56, 57]. Recent studies demonstrated that FOXO3a expression is regulated by the methylation status of its promoter in mouse embryonic fibroblasts [22]. The current studies indicated that the FOXO3a promoter is hypermethylated in breast cancer, and revealed that the methylation status of specific CpG sites (−365 and −365 bp) in the promoter region is pivotal in the epigenetic regulation of FOXO3a. FOXO3a has been identified as one of the target genes regulated by EZH2/H3K27me3/DNMTs-dependent transcriptional network [23, 58]. It is noted that DNMT1, DNMT3a, and DNMT3b are all recruited to the FOXO3 promoter in breast cancer HCC70 and MDA-MB-468 cells [23]. However, we found that knockdown of DNMT1, but not DNMT3a and DNMT3b, resulted in restoration of FOXO3a expression, indicating that the downregulation of FOXO3a is associated with DNMT1-mediated hypermethylation of its promoter in breast cancer. DNA demethylating agents have been shown to be effective in the treatment of hematological malignancies and several solid tumors [59]. Consistent with previous reports [60], we highlighted FOXO3a is an important target for demethylating agents. Upregulation of FOXO3a in breast cancer cells by demethylating agents can lead to effectively target BCSCs and inhibit tumor growth. Therefore, the FOXO3a level might be a marker for therapy response to demethylating agents. One of the most interesting findings in our study was that SOX2 feedback inhibited FOXO3a expression. We found that overexpression of SOX2 inhibited FOXO3a expression, which was completely abolished by transfection of DNMT1 siRNA. In glioblastome, SOX2 induces DNMT expression and methylation events that repress tumor suppressor miRNAs, which in turn promotes tumor propagation [32]. This supports our hypothesis that SOX2 directly transactivates DNMT1 expression and thereby alters the methylation landscape and feedback inhibits FOXO3a expression in breast cancer.

In summary, our findings demonstrate that DNMT1/FOXO3a/FOXM1/SOX2 signaling promotes BCSC properties, which might contribute to tumor initiation and progression in breast cancer, and that targeting this signaling is a potential therapeutic strategy for breast cancer.

## Materials and methods

### Cell culture

The normal breast mammary epithelial cell line MCF-10A, and human breast cancer cell line MCF-7, T47D, MDA-MB-231, BT549, BT474, SKBR3, and Hs578T were obtained from the American Type Culture Collection. Cell lines were reauthenticated by short tandem repeat analysis every 6 months after resuscitation in our laboratory. MCF-10A cells were cultured in Dulbecco’s Modified Eagle Medium (DMEM) (HyClone, Logan, UT, USA) supplemented with 5% horse serum, 20 μg/mL EGF, 0.5 μg/mL hydrocortisone, 0.1 μg/mL cholera toxin, 10 μg/mL insulin, and 1% penicillin/streptomycin. Breast cancer cells were cultured in DMEM containing 10% fetal bovine serum and 1% penicillin/streptomycin in a humidified incubator of 5% CO_2_ at 37 °C.

### Patients and specimens

Primary tumor specimens were obtained from 100 patients diagnosed with breast cancer who underwent complete resection in the Affiliated Tumor Hospital of Guangzhou Medical University between 2004 and 2008. Follow-up information was obtained from review of the patients’ medical record. Furthermore, twenty of fresh primary breast cancer tissues obtained from patients were used for real-time RT-PCR analysis. This study was approved by the Ethics Committee of Guangzhou Medical University and written informed consent was provided by all patients based on the Declaration of Helsinki.

### Bisulfite sequencing analysis

Genomic DNA from FFPE, fresh-frozen tissues, and cells was isolated using a QIAamp DNA FFPE Tissue Kit (Qiagen, Germany), AllPrep RNA/DNA Mini Kit (Qiagen), or EZ1 DNA Tissue Kit (Qiagen), respectively, according to the manufacturer’s instructions. An EpiTect Bisulfite Kit (Qiagen) was applied to conduct the bisulfite modification of DNA (1–2 mg). PyroMark Assay Design Software 2.0 (Qiagen) was used to design the bisulfite sequencing primers. The primer sequences are shown in Table S1. The PyroMark Q96 ID System and software (Qiagen) were utilized for the sequencing reaction and methylation level quantification.

### Immunohistochemical assay

Immunohistochemical assay was performed on formalin-fixed, paraffin-embedded sections of clinical breast cancer tissues or xenograft mice tissues. Briefly, the sections were deparaffinized in xylene, rehydrated with graded alcohol, and then boiled in 0.01 M citrate buffer (pH 6.0) for 20 min with an autoclave. Hydrogen peroxide (0.3%) was applied to block endogenous peroxide activity, and the sections were incubated with normal goat serum to reduce nonspecific binding. Tissue sections were incubated with the primary antibodies at 4 °C overnight. After incubation with the secondary antibody for 60 min, specimens were incubated with H_2_O_2_-diaminobenzidine until the desired stain intensity was developed. The antibodies used for IHC assays are shown in Table S2.

Sections were then counterstained with haematoxylin, dehydrated and mounted. Staining intensities and extents of FOXO3a, FOXM1, SOX2, and Dnmt1 expression were graded as follows: negative (score 0), weak (score 1), moderate (score 2), and strong (score 3). Percentage scores were assigned as 1, 1–25%; 2, 26–50%; 3, 51–75%; and 4, 76–100%. The scores of each tumor sample were multiplied to give a final score of 0–12, and the tumors were finally determined as negative (−), score 0; lower expression (+), score ≤ 4; moderate expression (++), score 5–8; and high expression (+++), score ≥ 9. All immunohistochemical staining were evaluated and scored by at least two independent pathologists. The cutoff score was chosen based on a measure of heterogeneity using the log-rank test statistical analysis with respect to overall survival. Receiver operating curve was used to determine the optimal cutoff score based on progression end point for FOXO3a, FOXM1, SOX2, and Dnmt1 expression. An optimal cutoff score was identified: a staining index of six or greater was used to define tumors of high expression, and five or lower for low expression.

### RNA interference and plasmid transfection

FOXO3a siRNAs (SignalSilence® FOXO3a siRNA I, Cat. No 6302; SignalSilence® FoxO3a siRNA II, Cat. No 6303) were purchased from Cell Signaling Technology (Denvers, MA, USA). FOXM1 siRNAs (FOXM1 siRNA I, Cat. No sc-270048; FOXM1 siRNA II, Cat. No sc-37615) were purchased from Santa Cruz Biotechnology Inc. (Santa Cruz, USA). DNMT1 siRNA, SOX2 siRNA were obtained from Ribbio (Guangzhou, China). The siRNA sequences are shown in Table S3. Expression plasmid for pCMV6-FOXO3a (Cat. No RC209846), pCMV6-SOX2 (Cat. No RC200757), and pCMV6-XL5 empty plasmid was purchased from Origene (Rockville, MD). For transient transfections, Lipofectamine 2000 reagent (Invitrogen) was used according to the manufacturer’s instructions. The cells were collected after transfection with siRNA oligonucleotides (100 nM) or plasmids (2 mg) for 48 h. The infection efficiency was validated using qRT-PCR or western blotting assays. To generate FOXO3a stably expressing cells, FOXO3a ORF was cloned into the lentiviral vector GV358 (GENECHEM, Shanghai, China). Using the packaging plasmids pHelper 1.0 and pHelper 2.0 (GENECHEM), lentivirus expressing FOXO3a was generated and used to infect breast cancer cells. After 72 h infection, infected cells were cultured in DMEM containing 1 μg/mL puromycin to select cells stably expressing FOXO3a. Cells that stably overexpressing FOXO3a were designated as MDA-MB-231/LV-FOXO3a, BT549/LV-FOXO3a. For shRNA experiments, short hairpin sequences against either the FOXO3a gene, or FOXM1 gene, or the scrambled shRNA sequences were cloned into the EGFP-labeled lentiviral vector GV248 (GENECHEM). The lentiviruses encoding FOXO3a shRNA, or FOXM1 shRNA were then generated and infected into cells as described above. The target sequences selected are shown in Table S3.

### Flow cytometry analysis

CD44-APC and CD24-PE antibodies (BD Pharmingen, San Diego, CA, USA) were used to fractionate the CD24^−^CD44^+^ population. Cells were harvested by dissociation using 0.05% trypsin/EDTA. A total of 1 × 10^6^ cells were resuspended in 200 μL HBSS with 2% FBS and then stained with the proper amount of antibodies (according to the instruction sheet) for 30 min at 4 °C. Cells incubated with unconjugated antibodies were stained with secondary antibodies for another 30 min at 4 °C. CD24^−^CD44^+^ population were assayed with flow cytometry (BD FACSAria III, BD Bioscience, USA).

For the ALDH assay, ALDH activity was monitored using ALDEFLUOR kit (Stemcell Technologies, Vancouver, BC, Canada) following the manufacturer’s instructions. Briefly, 10^6^ cells were suspended in 1 mL of assay buffer. Five microliter activated aldefluor substrate was added to the suspension, and an aliquot of 0.5 mL was immediately quenched with a specific ALDH inhibitor diethylaminobenzaldehyde. After incubation at 37 °C for 40 min, the cells were centrifuged and resuspended in 0.5 mL aldefluor assay buffer. ALDH^+^ cells were assayed with flow cytometry.

### Mammosphere formation assay

Cells were plated in ultralow attachment six-well plates at a low density of 1000 viable cells/mL. Cells were maintained in DMEM/F12 supplemented with B27, 20 ng/mL EGF, 20 ng/mL basic fibroblast growth factor, and 4 mg/mL heparin for 14 days. The mammospheres were photographed using inverted microscope (Leica, Hamburg, Germany).

### Soft agar assays

Soft agar assays were done by seeding cells at a density of 10^3^ in 60 mm culture dishes containing 0.3% top low-melt agarose and 0.5% bottom low-melt agarose. Cells were fed every 4 days, and colonies were stained with 0.2% p-iodonitrotetrazolium violet and counted after 2 weeks.

### Real-time RT-PCR

Total RNA was isolated using E.Z.N.A.® HP Total RNA Kit (Omega Bio-tek, Doraville, GA, USA). The reverse transcription was performed with the PrimeScript® RT reagent Kit (TakaRa, Shiga, Japan). After mixing the resulting complementary DNA template with PCR primers, respectively, and TaKaRa SYBR® Premix Ex Taq™, quantitative real-time PCR reaction was performed on ABI 7500 Fast Real-Time PCR System (Applied Biosystems, Foster City, CA). The PCR primers are shown in Supplemental Table S1. The relative levels of gene expression were represented as ΔCt-Ct gene-Ct reference, and the fold change of gene expression was calculated by the 2^−ΔΔCt^ method.

### Western blot

Total protein was isolated using RIPA buffer (Beyotime Biotechnology, China) that contained a protease inhibitor cocktail. Protein extracts were separated via 8–12% sodium dodecyl sulfatepolyacrylamide gel electrophoresis and transferred to polyvinylidene fluoride membranes. The membranes were subsequently blocked in 5% defatted milk and incubated with primary antibodies overnight at 4 °C. The species-matched secondary antibodies were then hybridized with the membranes at room temperature. Finally, the antigen–antibody reaction was visualized using enhanced chemiluminescence (Thermo, USA). The antibodies used for western blotting assays are shown in Supplementary Table 2.

### Immunofluorescence staining

Cells were fixed with 4% paraformaldehyde, permeabilized with PBS containing 0.1% Triton X-100 (PBS-T), and blocked with normal goat serum. Slides were blocked for 30 min with normal goat serum and incubated overnight at 4 °C with the anti-FOXM1 antibody (1:200 dilution) or anti-SOX2 antibody (1:200 dilution). After a wash step, slides were incubated with Alexa Fluor® 488 conjugate anti-rabbit (1:1000 dilution) or Alexa Fluor® 594 conjugate anti-rabbit IgG (1:1000 dilution) for 1 h, and then nuclei were stained with DAPI. The images were acquired using a Zeiss LSM710 confocal microscope (Zeiss, Germany).

### Chromatin immunoprecipitation

ChIP was performed using SimpleChIP® Plus Enzymatic Chromatin IP Kit (Cell Signaling Technology, Cat. No 9005) according to the manufacturer’s protocol. Briefly, cells were fixed with 1% formaldehyde for 10 min at RT. Next, the cells were washed twice with PBS at 4 °C, collected and resuspended in ice-cold lysis buffer, and lysed on ice for 30 min. The cells were homogenized on ice, to aid in nuclei release. Cells were sonicated five times for 5 s at 50% of maximal power (Fisher Sonic Dismembrator). The chromatin (25 μg) was immunoprecipitated for 12 h with 2 μg of specific antibodies against FOXO3a or FOXM1 or IgG and Protein G magnetic beads (25 μL). Beads were then washed sequentially for 5 min with the following buffers: ChIP Buffer I for one time and ChIP Buffer II for two times. The immune complexes were eluted with 50 μL elution buffer AM2. The supernatants were reverse cross-linked by heating at 65 °C for 12 h, treated with 1 μL RNaseA at 37 for 15 min, and digested with 2 μL proteinase K at 37 °C for 1 h. DNA was obtained by phenol and phenol/chloroform extractions. The percentage of chromatin-bound recovered DNA was quantified against DNA input. Primers used for the amplification of the precipitated DNA are listed in Supplementary Table S1.

### Dual-luciferase reporter assays

Wild-type SOX2 promoter regions (−1960to −1 bp) contain the FOX binding sites or mutant of SOX2 promoter regions were subcloned into pGL3 vector. The FOXO3a promoter construct (−500 to +1 bp) were subcloned into pCpGfree-Luc vector using Rapid DNA Ligation Kit (Thermo Scientific). Point mutations at CpG sites in the FOXO3a promoter constructs were generated by converting CG to TG using the QuickChange II Site-Directed Mutagenesis Kit (Agilent Technologies). All constructs were verified by sequencing. For the luciferase reporter assay, cells were plated in 12-well plates for 24 h and transfected with luciferase reporter constructs and pRL-TK Renilla luciferase. Cells were harvested and luciferase activity was measured 48 h later using the Dual-Luciferase Reporter Assay system (Promega) according to the manufacturer’s instructions. Briefly, cells were collected and washed with PBS. Passive lysis buffer (Promega) 500 μL per well was added with gentle rocking for 15 min at RT. Ten microlitres of lysate were transferred in black 96-well plate (Thermo). Firefly and Renilla luciferase activity were assayed sequentially to the cell lysate in each well. Transcriptional activity was calculated as the ratio of firefly luciferase activity (reporter) to Renilla luciferase activity (control).

### Animal studies

All animal work was performed in accordance with protocols approved by the Animal Experimentation Ethics Committee of Guangzhou medical University.

To evaluate the effect of FOXO3a on stemness, limiting dilution assays were performed in nude mice. Cells were injected subcutaneously into 4-week-old female nude mice at indicated cell concentrations per site. Six mice were used in each experimental group. Tumor formation was checked every 3 days and the observation time was 3 weeks in total. The frequency of TICs was calculated using the extreme limiting dilution analysis program (http://bioinf.wehi.edu.au/software/elda/) [61].

To evaluate the effect of 5-AzaC on tumor growth, 5 × 10^5^ MDA-MB-231 cells or BT549 cells were subcutaneously into the nude mice. When tumors became visible (~3 × 3 mm in size), the mice were randomly divided into two groups of four animals and treated intraperitoneally with 5-AzaC at a dose of 0.5 mg/kg body weight every other day for 4 weeks, whereas the control group was treated with an equivalent volume of normal saline. Tumor size and body weight were measured every 3 days. The tumor volume was calculated using the formula: *V* = 1/2 × larger diameter × (smaller diameter)^2^, and growth curves were plotted using average tumor volume within each experimental group at the set time points. At the end of treatment, the animals were killed, and the tumors were removed and weighed for use in immunohistochemical staining or western blot studies.

### Statistical analysis

Statistical analyses were conducted using the SPSS16.0 software. Comparisons between groups were analyzed by the *t* test and *χ*2 test. Overall survival curves were plotted according to the Kaplan–Meier method with the log-rank test applied for comparison. Survival was measured from the day of surgery. Variables with values of *P* < 0.05 by univariate analysis were used in subsequent multivariate analysis based on the Cox proportional hazards model. The differences were considered statistically significant at *P* < 0.05.

## Supplementary information

Supplementary Figure S1–S12

Table S1–S5
