# Supplementary material for: Downregulation of FOXO3a by DNMT1 promotes breast cancer stem cell properties and tumorigenesis
Source: Cell Death Differ. 2019 Jul 11;27(3):966–83. doi: 10.1038/s41418-019-0389-3 (PMC7206060; doi:10.1038/s41418-019-0389-3)
Supplement: Supplementary file 1 — Supplementary Figure S1–S12 [file 41418_2019_389_MOESM1_ESM.docx]

**Supplementary Figure S1-S12**


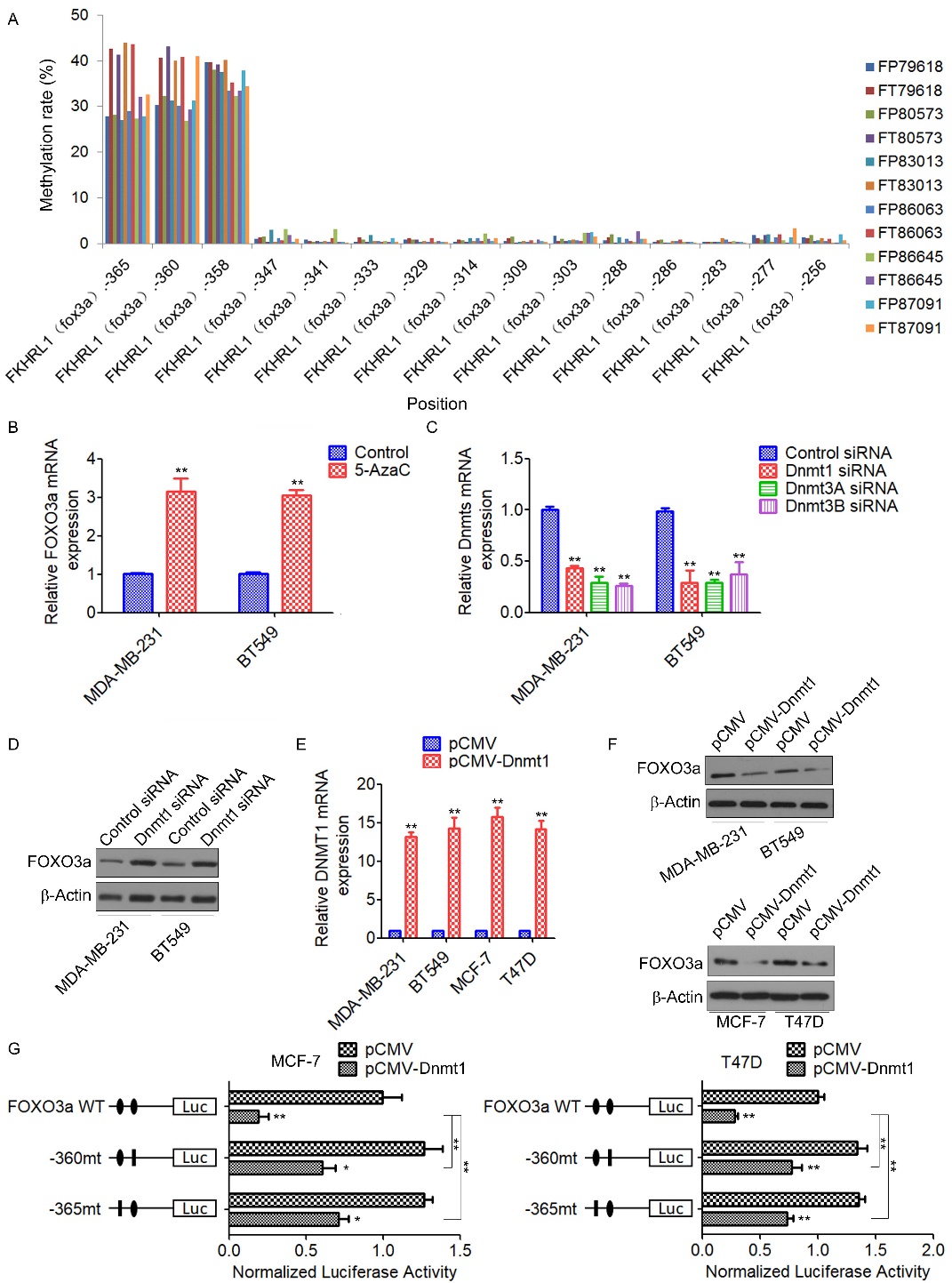


**Figure S1. DNMT1 suppresses the expression of FOXO3a in breast cancer**. (A) Bisulfite sequencing analysis of the FOXO3a promoter region and the average methylation levels in normal tissues (FP, n=6) and breast cancer tissues (FT, n=6). (B) MDA-MB-231 and BT549 cells were treated with 5-AzaC for 48 h, FOXO3a expression was measured by qRT-PCR analysis. (C) MDA-MB-231 and BT549 cells were transfected with DNMTs siRNA for 48 h, DNMTs mRNA expression was measured by qRT-PCR analysis. (D) MDA-MB-231 and BT549 cells were transfected with DNMT1 siRNA, FOXO3a expression was measured by Western blot. (E-F) Breast cancer cells were transfected with DNMT1 expression vector for 48 h, (E) DNMT1 mRNA expression was measured by qRT-PCR analysis; (F) FOXO3a expression was measured by Western blot. (G) DNMT1 expression vector and FOXO3a wild type promoter constructs or promoter constructs containing site-specific CpG mutations were co-transfected into MCF-7 and T47D cells. Assessment of activities of FOXO3a promoter constructs containing different mutations was performed by luciferase assay. Point mutations (CG to TG) were created at CpG sites located at −365-bp and −360-bp. A two-tailed Student’s t-test was used for statistical analysis (*P < 0.05, **P < 0.01).


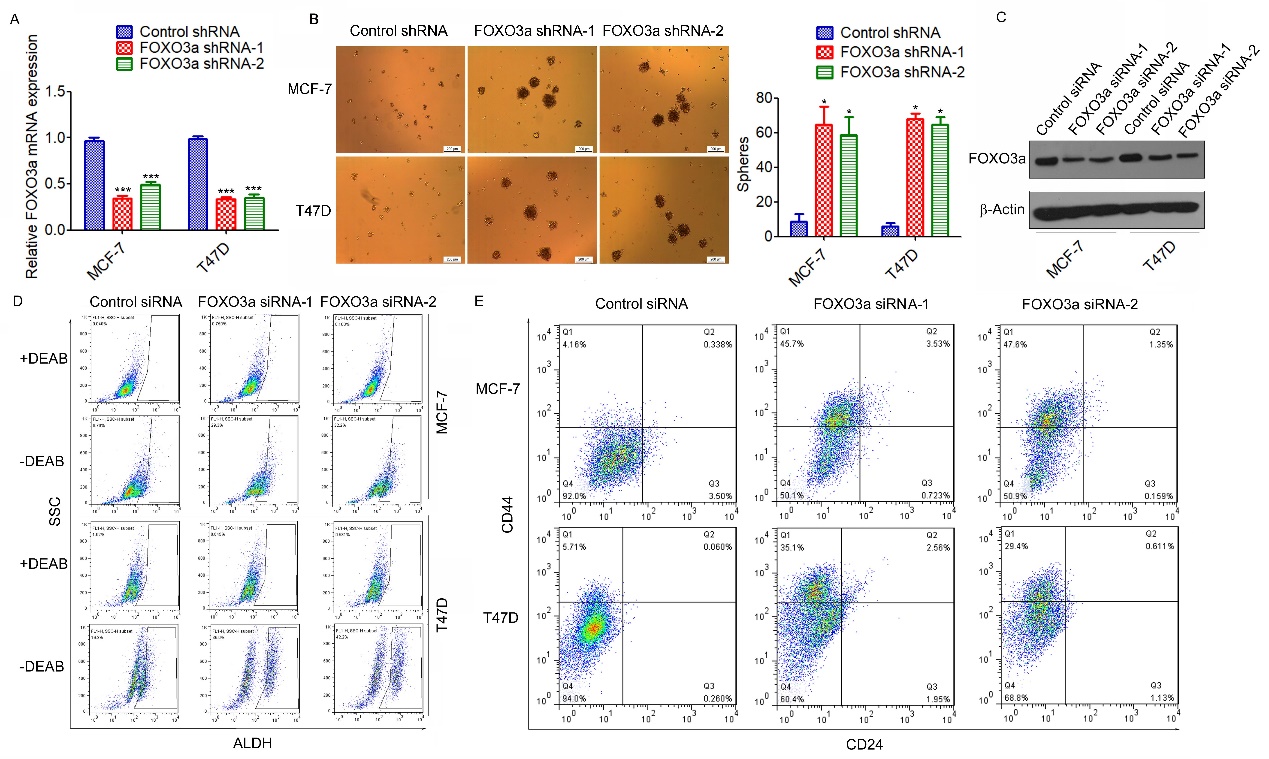


**Figure S2.** **Knockdown of FOXO3a promotes breast cancer stem cell properties in MCF-7 and T47D cells.** (A) MCF-7 and T47D cells were transfected with FOXO3a shRNA, (A) FOXO3a expression was measured by qRT-PCR analysis; (B) Tumorsphere formation assay was performed to evaluate self-renewal of CSCs in control and FOXO3a-knockdown cells. (C-E) MCF-7 and T47D cells were transfected with FOXO3a siRNA, (C) FOXO3a expression was measured by Western blot; (D) the percentage of CD44^high^/CD44^low^ cells was measured using flow cytometry; (E) the percentage of ALDH^+^ cells was measured by ALDEFLUOR Assay. A two-tailed Student’s t-test was used for statistical analysis (*P < 0.05, ***P < 0.001).


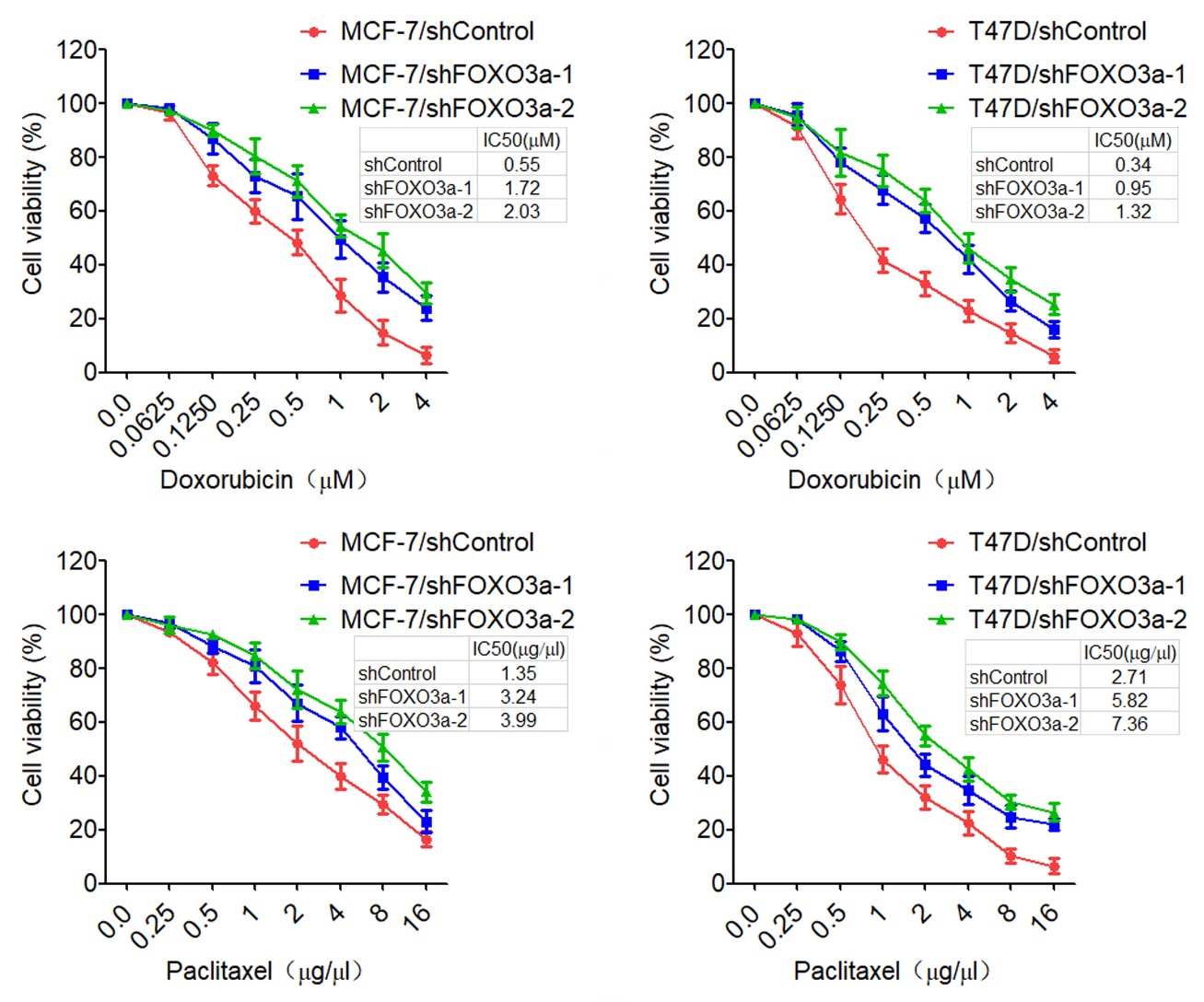


**Figure S3.** Downregulation of FOXO3a expression enhanced drug resistance in breast cancer. MCF-7 and T47D transfected with FOXO3a shRNA or Control shRNA were treated with doxorubicin or paclitaxel at indicated concentrations for 72 h, cell viability was evaluated by MTS assay. Table indicates the IC50 values for control or FOXO3a-knockdown cells. (*P < 0.05, **P < 0.01).


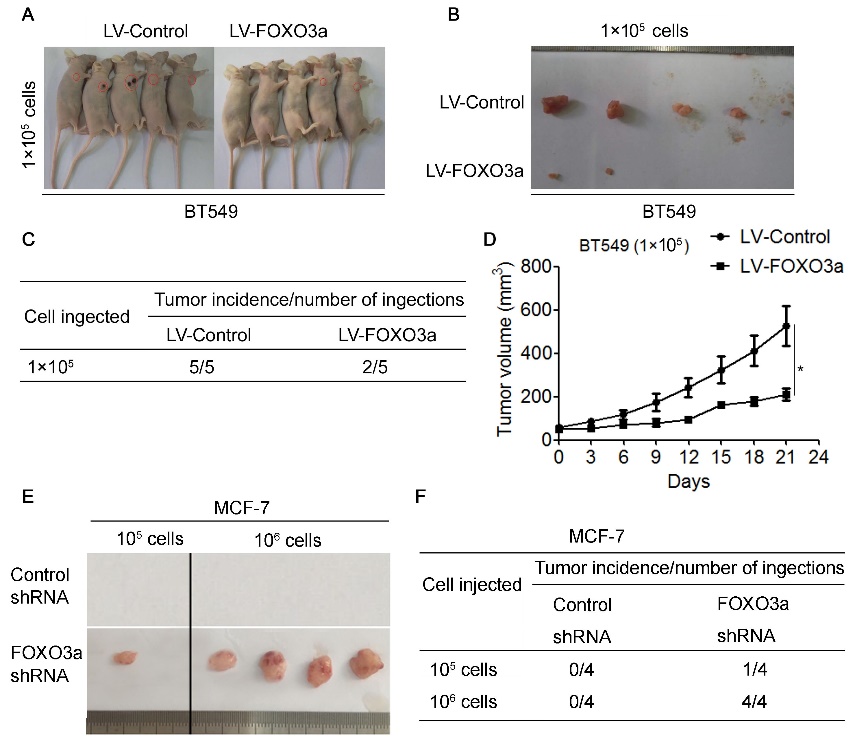


**Figure S4. FOXO3a impairs tumorigenicity and tumor growth in Vivo.** (A-D) 1×10^5^ BT549/Control and BT549/FOXO3a cells were implanted in nude mice (n=5 per group), (A) Representative images of the tumors are shown; (B) At the experimental endpoint, the tumors were dissected and imaged as indicated; (C) Tumor formation frequencies for different numbers of the indicated cells; (D) The tumor volume was measured on the indicated days. (E-F) 1×10^6^ and 1×10^5^ MCF-7/Control shRNA and MCF-7/FOXO3a shRNA cells were implanted in nude mice (n=4 per group), (E) At the experimental endpoint, the tumors were dissected and imaged as indicated; (F) Tumor formation frequencies for different numbers of the indicated cells. (*P < 0.05).


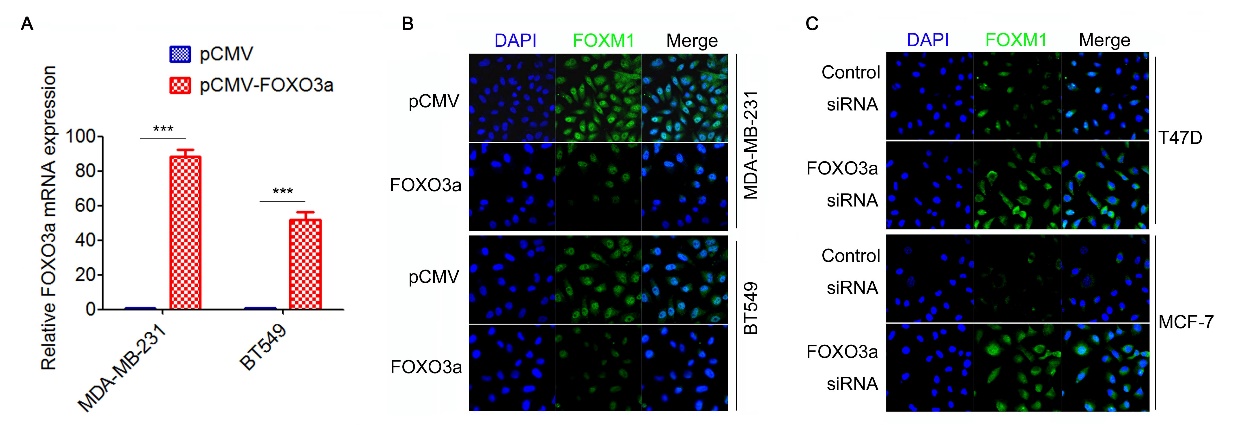


**Figure S5. FOXO3a suppresses the expression FOXM1 in breast cancer. (A)** MDA-MB-231 and BT549 cells were transfected with FOXO3a-expression vector, FOXO3a expression was measured by qRT-PCR. (B) MDA-MB-231 and BT549 cells were transfected with FOXO3a-expression vector, FOXM1 expression was measured by Immunofluorescence staining. (C) T47D and MCF-7 cells were transfected with FOXO3a siRNA, FOXM1 expression was measured by immunofluorescence staining. A two-tailed Student’s t-test was used for statistical analysis (***P < 0.001).


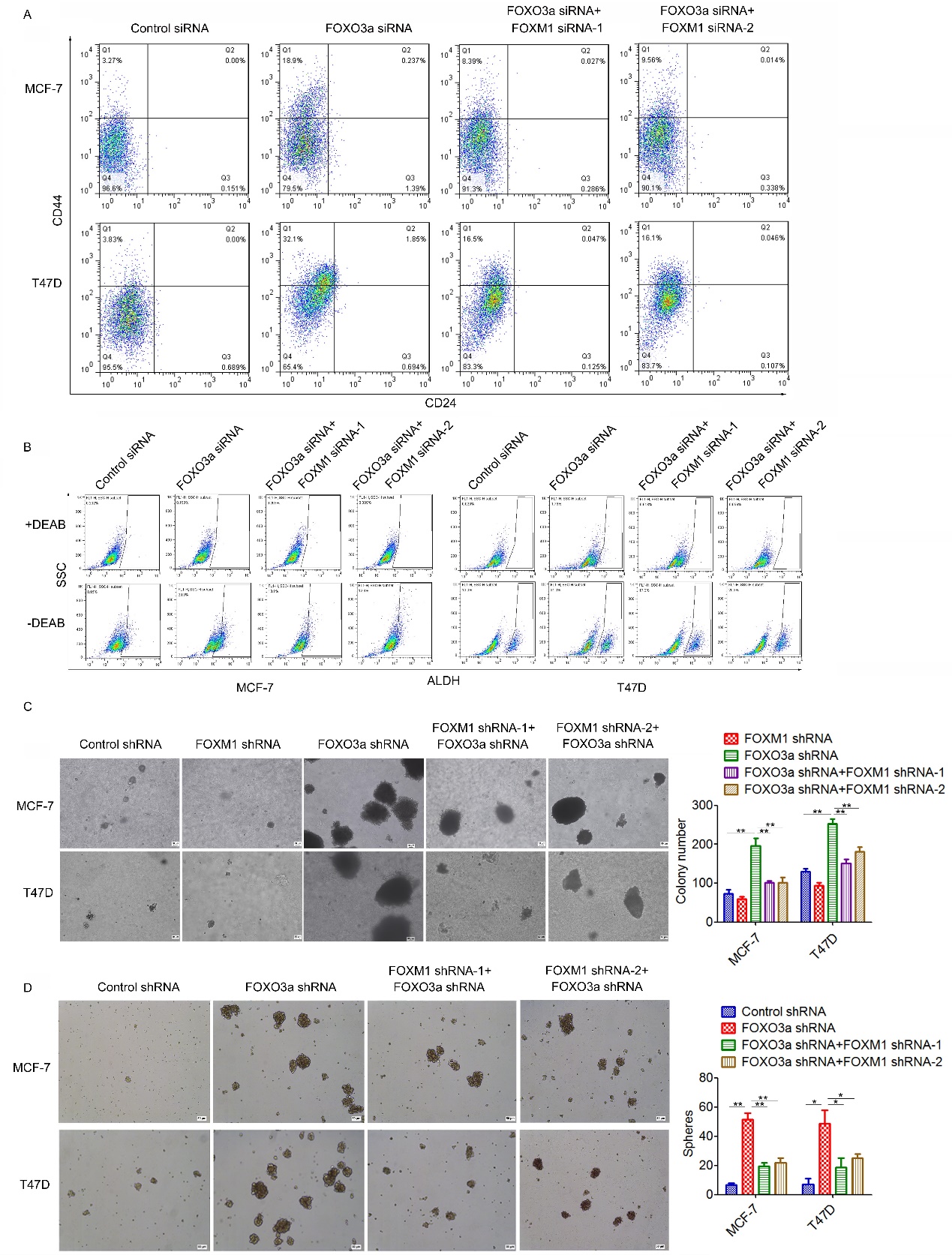


**Figure S6. FOXO3a suppresses breast cancer stem cell properties via inhibition of FOXM1.** (A-B) MCF-7 and T47D cells were transfected with FOXO3a siRNA plus FOXM1 siRNA, (A) The percentage of CD44high/CD44low cells was measured using flow cytometry; (B) The percentage of ALDH+ cells was measured by ALDEFLUOR Assay. (C-D) MCF-7 and T47D cells were transfected with FOXO3a shRNA plus FOXM1 shRNA, (C) enhanced mammosphere formation capacity and (D) anchorage-independent growth induced by FOXO3a shRNA could be reversed by FOXM1 shRNA. A two-tailed Student’s t-test was used for statistical analysis (*P < 0.05, **P < 0.01).


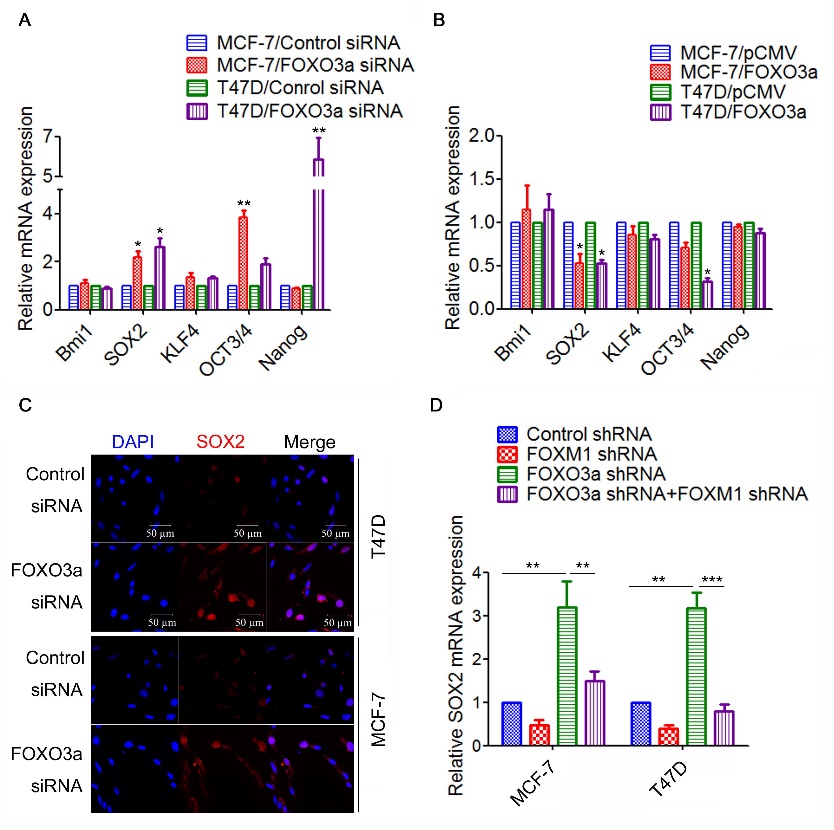


**Figure S7. FOXO3a inhibited SOX2 expression via FOXM1-dependant manner.** (A) MCF-7 and T47D cells were transfected with FOXO3a siRNA, the expressions of stemness-related genes were measured by qRT-PCR. (B) MCF-7 and T47D cells were transfected with FOXO3a-expression vector, the expressions of stemness-related genes were measured by qRT-PCR. (C) T47D and MCF-7 cells were transfected with FOXO3a siRNA, FOXM1 expression was measured by Immunofluorescence staining. (D) MCF-7 and T47D cells were transfected with FOXO3a shRNA plus FOXM1 shRNA, SOX2 expression was measured by qRT-qPCR. A two-tailed Student’s t-test was used for statistical analysis (*P < 0.05, **P < 0.01, ***P < 0.001).


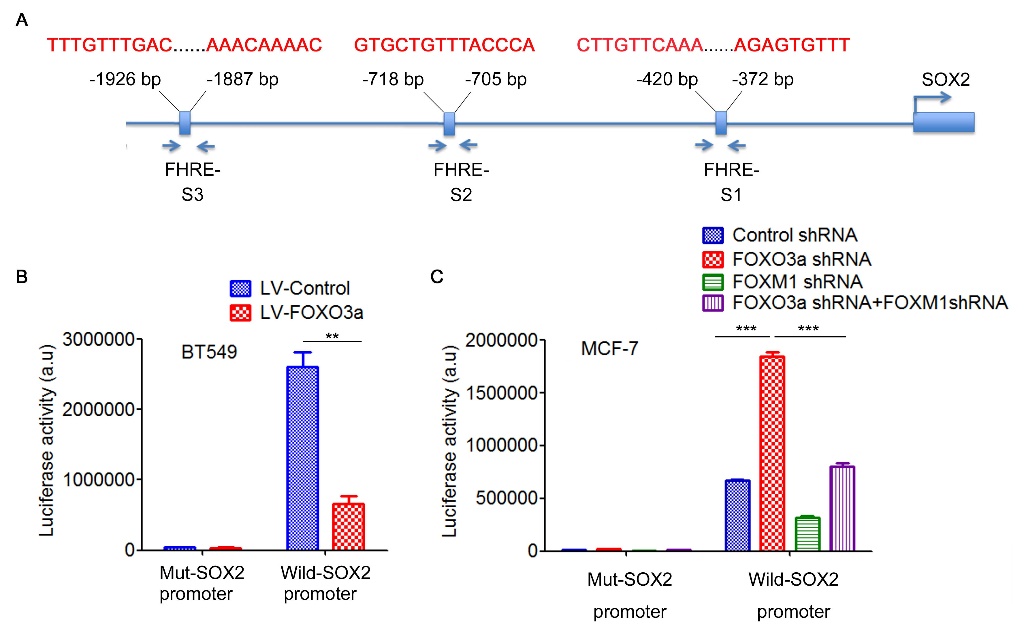


**Figure S8.** **FOXO3a inhibits the activity of SOX2 promoter.** (A) A sequence analysis of the promoter regions revealed three conserved FOX-binding sites at the core promoter region of SOX2 (FHRE-S1, FHRE-S2, FHRE-S3). (B) BT549 cells were transfected with FOXO3a expression vector plus a luciferase reporter construct containing the indicated promoter regions or mutant promoter regions. Relative luciferase activities were measured 48 h after transfection. Firefly luciferase activity of the reporter construct was normalized to internal Renilla luciferase activity. (C) MCF-7 cells were transfected with FOXO3a shRNA or/and FOXM1 shRNA plus a luciferase reporter construct containing the indicated promoter regions or mutant promoter regions. Relative luciferase activities were measured 48 h after transfection. A two-tailed Student’s t-test was used for statistical analysis (**P < 0.01, ***P < 0.001).


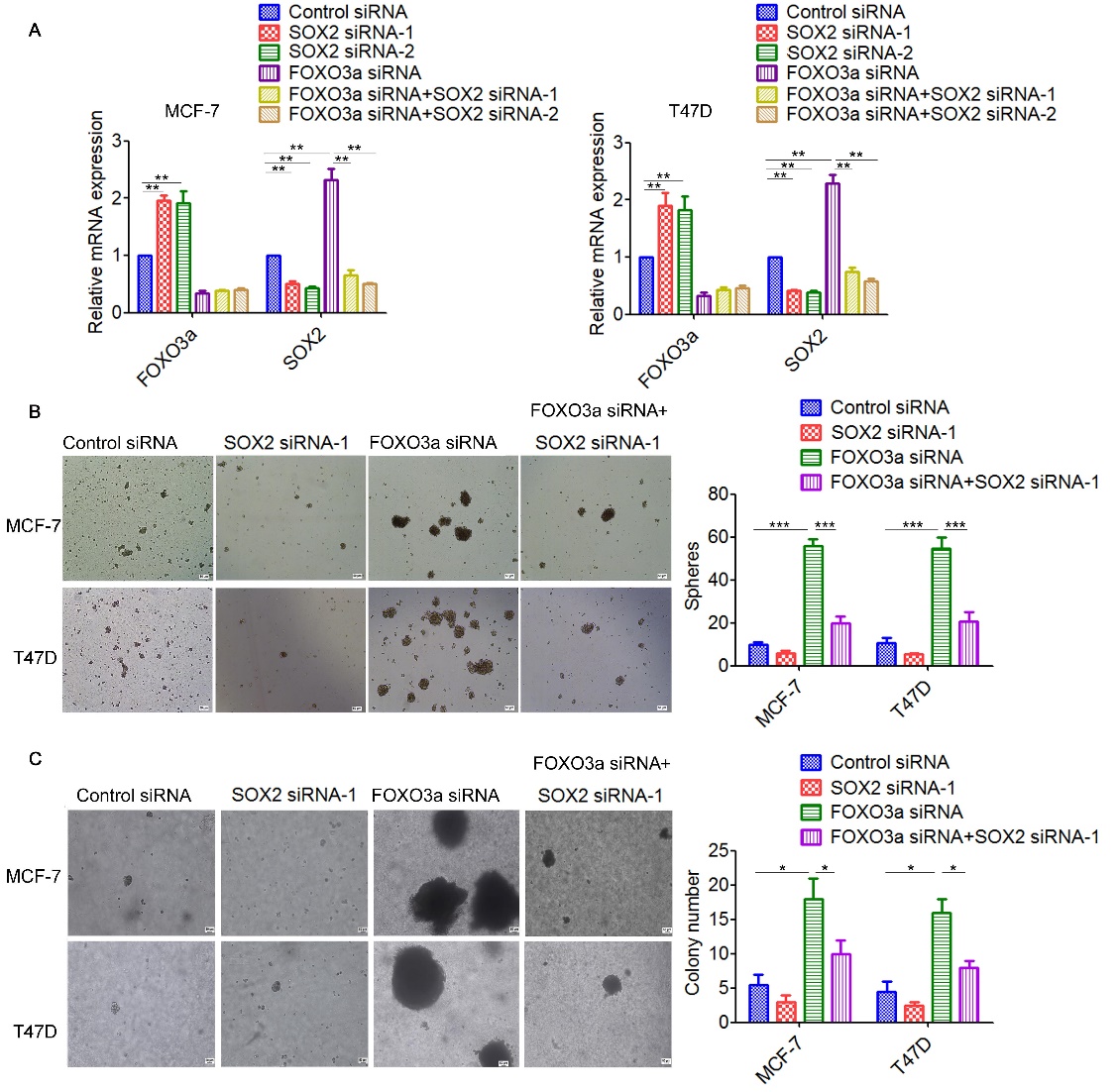


**Figure S9. FOXO3a /FOXM1 axis regulates BCSC properties in SOX2-dependent mechanism.** MCF-7 and T47D cells were transfected with FOXO3a shRNA plus SOX2 siRNA, (A) FOXO3a, and SOX2 expression was measured by qRT-qPCR. (B) Enhanced mammosphere formation capacity and (C) anchorage-independent growth induced by FOXO3a shRNA could be reversed by SOX2 siRNA. A two-tailed Student’s t-test was used for statistical analysis (*P < 0.05, **P < 0.01, ***P < 0.001).


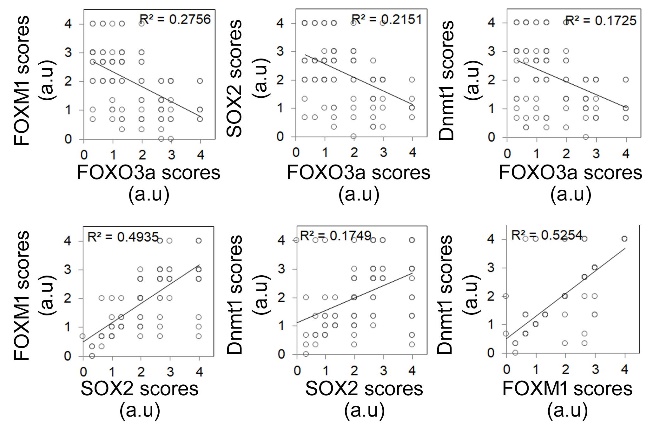


**Figure S10. Clinical correlations among FOXO3a, FOXM1, SOX2 and DNMT1 in breast cancer samples.**


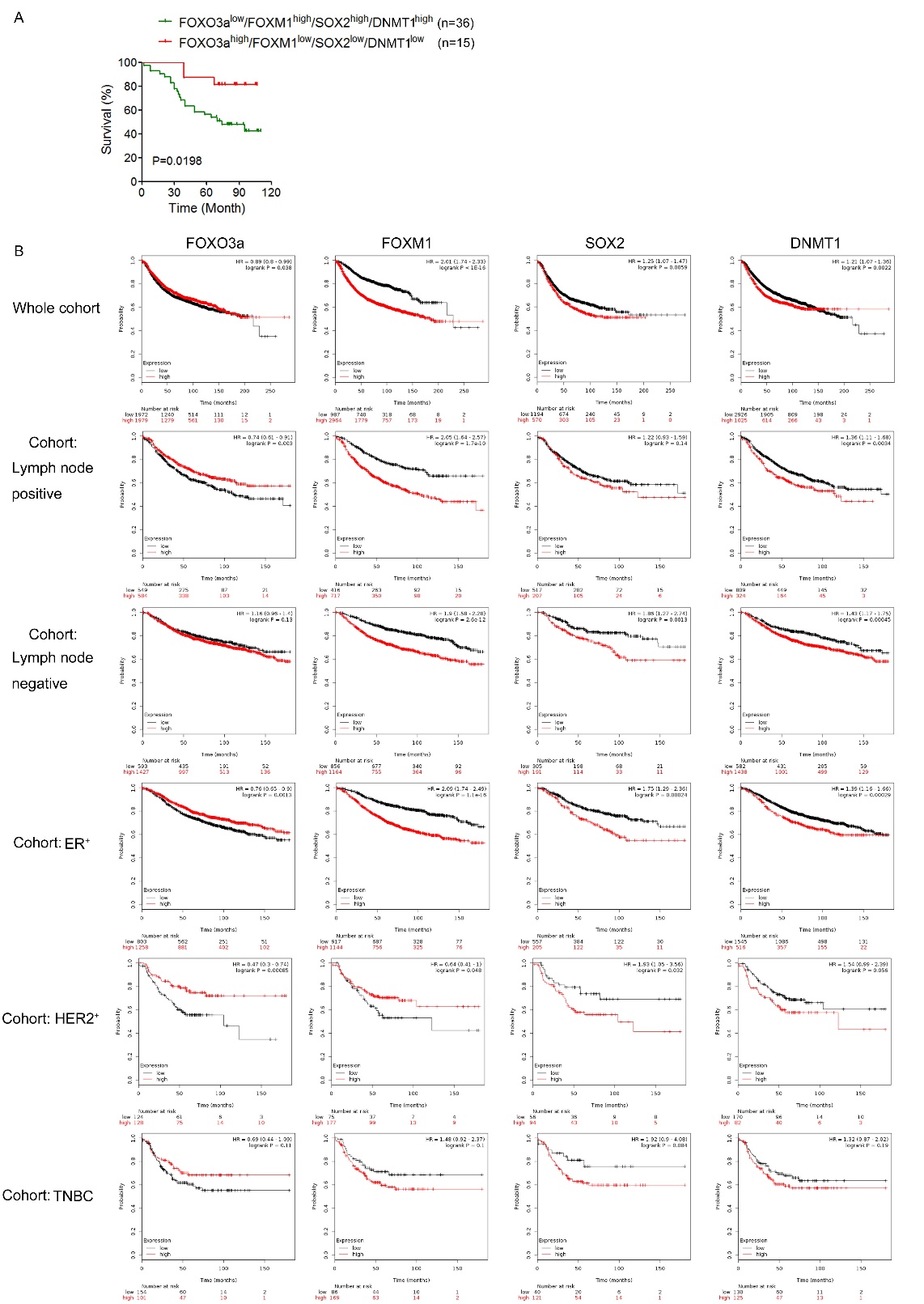


**Figure S11. Dysregulation of DNMT1/FOXO3a/FOXM1/SOX2 signaling exhibits clinical significance**. (A) Survival curves of breast cancer patients with combined use of four biomarkers FOXO3a, FOXM1, SOX2 and DNMT1. (B) Kaplan–Meier OS curves (<http://kmplot.com/analysis/>) of 6234 breast cancer patients relative to different expression levels of FOXO3a (probe 201590_x_at), FOXM1(probe 202580_x_at), SOX2 (probe 228038_x_at), and DNMT1 (probe 201697_x_at).


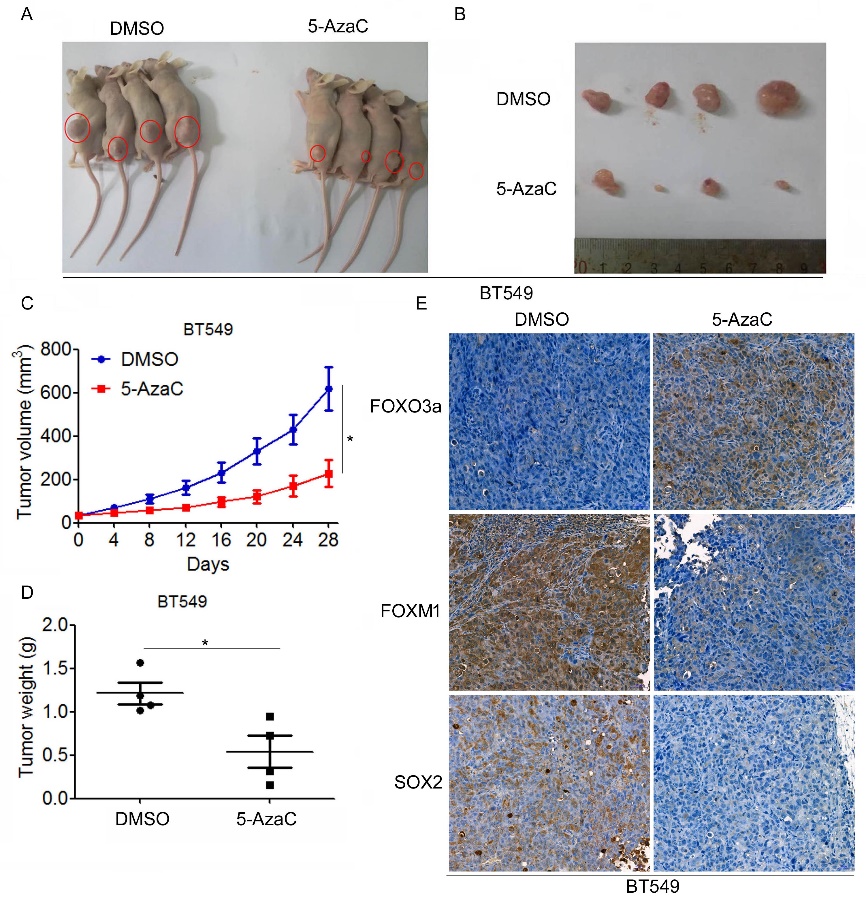


**Figure S12. Inhibition of DNMT activity suppresses tumorigenesis and tumor growth in BT549 cells.** (A-D) BT549 cells were implanted in nude mice and palpable tumors were allowed to develop for 7 days. 5-AzaC at a dose of 0.5 mg/kg body weight was injected every other day for 4 weeks. (A) Representative images of the tumors are shown. (B) At the end of treatment, tumors were excised and subjected to further analyses. (C) Tumor size was measured at indicated time intervals, tumor volumes in 5-AzaC-treated animals were smaller than that of control mice. (D) 5-AzaC treatment resulted in significantly lower tumor weight compared with control. (E) Tumor tissues derived from control and 5-AzaC -treated mice were resected, fixed, sectioned, and placed on slides. Tumor specimens were subjected to immunohistochemical staining with antibodies specific to FOXO3a, FOXM1, and SOX2. A two-tailed Student’s t-test was used for statistical analysis. (*P < 0.05).
