# Supplementary material for: Downregulation of FOXO3a by DNMT1 promotes breast cancer stem cell properties and tumorigenesis
Source: Cell Death Differ. 2019 Jul 11;27(3):966–83. doi: 10.1038/s41418-019-0389-3 (PMC7206060; doi:10.1038/s41418-019-0389-3)
Supplement: Supplementary file 2 — Table S1–S5 [file 41418_2019_389_MOESM2_ESM.docx]

**Supplementary Table S1. Primers used in this study.**

| Gene | Sequence (5’-3’) |
| --- | --- |
| qRT-PCR primer | |
| FOXO3a-F | GCGTGCCCTACTTCAAGGATAAG |
| FOXO3a-R | GACCCGCATGAATCGACTATG |
| GAPDH-F | CACCCAGAAGACTGTGGATGG |
| GAPDH-R | GTCTACATGGCAACTGTGAGG |
| FOXM1-F | TGCAGCTAGGGATGTGAATCTTC |
| FOXM1-R | GGAGCCCAGTCCATCAGAACT |
| SOX2-F | CCCACCTACAGCATGTCCTACTC |
| SOX2-R | TGGAGTGGGAGGAAGAGGTAAC |
| OCT4-F | TTCAGCCAAACGACCATCTG |
| OCT4-R | CACGAGGGTTTCTGCTTTGC |
| Nanog-F | TTCCCTCCTCCATGGATCTG |
| Nanog-R | TGTTTCTTGACTGGGACCTTGTC |
| Bmi-F | CTGCCAATGGCTCTAATGAA |
| Bmi-R | TTCCGATCCAATCTGTTCTG |
| KLF4-F | TCTCAAGGCAGACCTGCGAA |
| KLF4-R | TAGTGCCTGGTCAGTTCATC |
| CD44-F | CCTCTTGGCCTTGGCTTTG |
| CD44-R | TCCATTGCCACTGTTGATCAC |
| ALDH1-F | TCGTCTGCTGCTGGCGACAATG |
| ALDH1-R | CCCAACCTGCACAGTAGCGCAA |
| DNMT1-F | AAGACAAAGACCAGGATGAGAAG |
| DNMT1-R | GGGTGTTGGTTCTTTGGTTTG |
| DNMT3A-F | TATTGATGAGCGCACAAGAGAGC |
| DNMT3A-R | GGGTGTTCCAGGGTAACATTGAG |
| DNMT3B-F | CCATTCGAGTCCTGTCATTG |
| DNMT3B-R | GCAATGGACTCCTCACACAC |
| BSP sequencing primer | |
| F1 | TAGTCTGAGTCTGCTGGTTAGAG |
| R1 | AGGAAAACAACACAAACTTAAA |
| ChIP-PCR primers | |
| SOX2 site1-F | CGTCACATGGATGGTTGTCTATTAACTTGTTCA |
| SOX2 site1-R | CTCTCAGTCCTAGTCTTAAAGAGGCAGC |
| SOX2 site2-F | ACGTAGTCTTAGTGCTGTTTACCCACT |
| SOX2 site2-R | GAGGAGGCTCCCAGAGCC |
| SOX2 site3-F | CTTTGTTTGACTCCGTGTAGCGACA |
| SOX2 site3-R | CTTTGTTTGACTCCGTGTAGCGACA |
| Control site-F | TACTAGCGGTTTTACGGGCG |
| Control site-R | TCGAACAGGAGGAGCAGAGAGCGA |

**Supplementary Table S2. Antibodies used in this study.**

| **Antibody name** | **Source** | **Manufacturer** | **Catalog no.** | **Working concentration** |
| --- | --- | --- | --- | --- |
| FOXO3a | Rabbit | Cell Signaling Technology | #2497 | WB, 1:1000 |
| FOXO3a | Rabbit | Abcam | # ab12162 | IHC, 1:200  CHIP, 1:100 |
| FOXM1 | Rabbit | Abcam | # ab180710 | WB, 1:1000  IHC, 1:100 |
| FOXM1 | Rabbit | Cell Signaling Technology | #5436 | WB, 1:1000  IF, 1:100 |
| FOXM1 | Rabbit | Cell Signaling Technology | #20459 | CHIP, 0.2mg per 2ml Protein A/G sepharose beads |
| SOX2 | Rabbit | Cell Signaling Technology | #3579 | WB, 1:1000  IF, 1:200 |
| SOX2 | Rabbit | Abcam | #ab171380 | IHC, 1:100 |
| OCT4 | Rabbit | Cell Signaling Technology | #2750 | WB, 1:1000 |
| Nanog | Rabbit | Cell Signaling Technology | #4903 | WB, 1:1000 |
| β-actin | Mouse | Cell Signaling Technology | #3700 | WB, 1:2000 |

**Supplementary Table S3. siRNA sequences used in this study.**

| Gene | siRNA Sequence |
| --- | --- |
| Control siRNA | UUCUCCGAACGUGUCACGUTT |
| DNMT1 siRNA | CAATGAGACTGACATCAAA |
| DNMT3A siRNA | GCCTGGAGCCACCAGAAGA |
| DNMT3B siRNA | GCAACGATCTCTCAAATGT |
| SOX2 siRNA-1 | CCACCTACAGCATGTCCTA |
| SOX2 siRNA-2 | GGAGCACCCGGATTATAAA |
| Control shRNA | GCTTCGCGCCGTAGTCTTA |
| FOXO3a shRNA-1 | GCTCACTTCGGACTCACTTAG |
| FOXO3a shRNA-2 | GGAACGTGATGCTTCGCAATG |
| FOXM1 shRNA-1 | GGACCACTTTCCCTACTTT |
| FOXM1 shRNA-2 | CGGAAATGCTTGTGATTCA |

**Supplementary Table S4. Analysis of the correlation between the expression of FOXO3a in primary breast cancer and its clinicopathological parameters.**

| Characteristics | Number of patients (n=100) | FOXO3a expression | | *P*-value |
| --- | --- | --- | --- | --- |
|  |  | Low (n=59) | High (n=41) |  |
| Age | | | | |
| <50 (years) | 40 | 22 | 18 | 0.5067 |
| >50 (years) | 60 | 37 | 23 |  |
| Histological grade | | | | |
| 1 | 21 | 7 | 14 | 0.0268 |
| 2 | 50 | 33 | 17 |  |
| 3 | 29 | 19 | 10 |  |
| Stage | | | | |
| I | 10 | 2 | 8 | 0.0211 |
| II | 72 | 44 | 28 |  |
| III-IV | 18 | 12 | 5 |  |
| Lymph node metastasis | | | | |
| Negative | 53 | 23 | 30 | <0.01 |
| Positive | 47 | 36 | 11 |  |
| Molecular subtype | | | | |
| ER+ | 51 | 20 | 31 | <0.01 |
| HER2+ | 19 | 12 | 7 |  |
| TNBC | 30 | 27 | 3 |  |

**Supplementary Table S5. Analysis of the correlation between the expression of FOXM1/SOX2/Dnmt1 in primary breast cancer and its clinicopathological parameters.**

| Characteristics | Number of patients (n=100) | FOXM1 expression | | *P*-value | SOX2  expression | | *P*-value | Dnmt1  Expression | | *P*-value |
| --- | --- | --- | --- | --- | --- | --- | --- | --- | --- | --- |
|  |  | Low (n=37) | High (n=63) |  | Low (n=28) | High (n=72) |  | Low (n=39) | High (n=61) |  |
| Age | | | | | | | | | | |
| <50 (years) | 40 | 13 | 27 | 0.4468 | 12 | 28 | 0.7161 | 12 | 28 | 0.1319 |
| >50 (years) | 60 | 24 | 36 |  | 16 | 44 |  | 27 | 33 |  |
| Histological grade | | | | | | | | | | |
| 1 | 21 | 12 | 9 | 0.0129 | 11 | 10 | 0.0197 | 13 | 8 | 0.0516 |
| 2 | 50 | 20 | 30 |  | 11 | 39 |  | 17 | 33 |  |
| 3 | 29 | 5 | 24 |  | 6 | 23 |  | 9 | 20 |  |
| Stage | | | | | | | | | | |
| I | 10 | 7 | 3 | <0.01 | 8 | 2 | <0.01 | 8 | 2 | <0.01 |
| II | 72 | 23 | 49 |  | 14 | 58 |  | 27 | 45 |  |
| III-IV | 18 | 7 | 11 |  | 6 | 12 |  | 4 | 14 |  |
| Lymph node metastasis | | | | | | | | | | |
| Negative | 53 | 29 | 24 | <0.01 | 20 | 33 | 0.0213 | 32 | 21 | <0.01 |
| Positive | 47 | 8 | 39 |  | 8 | 39 |  | 7 | 40 |  |
| Molecular subtype | | | | | | | | | | |
| ER+ | 51 | 26 | 25 | <0.01 | 21 | 30 | <0.01 | 20 | 31 | 0.9293 |
| Her2+ | 19 | 3 | 16 |  | 5 | 14 |  | 8 | 11 |  |
| TNBC | 30 | 8 | 22 |  | 2 | 28 |  | 11 | 19 |  |
